# Chronic pain and local pain in usually painless conditions including neuroma may be due to compressive proximal neural lesion

**DOI:** 10.3389/fpain.2023.1037376

**Published:** 2023-02-20

**Authors:** Valdas Macionis

**Affiliations:** Independent Researcher, Vilnius, Lithuania

**Keywords:** chronic pain, nociceptive sensitization, nerve compression syndromes, nervous system trauma, neuroma, neuropathic pain, nociception, pain management and treatment

## Abstract

It has been unexplained why chronic pain does not invariably accompany chronic pain-prone disorders. This question-driven, hypothesis-based article suggests that the reason may be varying occurrence of concomitant peripheral compressive proximal neural lesion (cPNL), e.g., radiculopathy and entrapment plexopathies. Transition of acute to chronic pain may involve development or aggravation of cPNL. Nociceptive hypersensitivity induced and/or maintained by cPNL may be responsible for all types of general chronic pain as well as for pain in isolated tissue conditions that are usually painless, e.g., neuroma, scar, and Dupuytren's fibromatosis. Compressive PNL induces focal neuroinflammation, which can maintain dorsal root ganglion neuron (DRGn) hyperexcitability (i.e., peripheral sensitization) and thus fuel central sensitization (i.e., hyperexcitability of central nociceptive pathways) and a vicious cycle of chronic pain. DRGn hyperexcitability and cPNL may reciprocally maintain each other, because cPNL can result from reflexive myospasm-induced myofascial tension, muscle weakness, and consequent muscle imbalance- and/or pain-provoked compensatory overuse. Because of pain and motor fiber damage, cPNL can worsen the causative musculoskeletal dysfunction, which further accounts for the reciprocity between the latter two factors. Sensitization increases nerve vulnerability and thus catalyzes this cycle. Because of these mechanisms and relatively greater number of neurons involved, cPNL is more likely to maintain DRGn hyperexcitability in comparison to distal neural and non-neural lesions. Compressive PNL is associated with restricted neural mobility. Intermittent (dynamic) nature of cPNL may be essential in chronic pain, because healed (i.e., fibrotic) lesions are physiologically silent and, consequently, cannot provide nociceptive input. Not all patients may be equally susceptible to develop cPNL, because occurrence of cPNL may vary as vary patients' predisposition to musculoskeletal impairment. Sensitization is accompanied by pressure pain threshold decrease and consequent mechanical allodynia and hyperalgesia, which can cause unusual local pain via natural pressure exerted by space occupying lesions or by their examination. Worsening of local pain is similarly explainable. Neuroma pain may be due to cPNL-induced axonal mechanical sensitivity and hypersensitivity of the nociceptive *nervi nervorum* of the nerve trunk and its stump. Intermittence and symptomatic complexity of cPNL may be the cause of frequent misdiagnosis of chronic pain.

## Introduction

Why are some neuromas painful, while others are painless? Why do some small articular fractures result in a very painful osteoarthritis, while some disastrous articular injuries may go asymptomatic? Similar questions can be asked regarding common ganglia of the hand, rheumatoid arthritis, Dupuytren's fibromatosis, and scars. It is also puzzling why some patients with systemic diseases develop chronic pain, while others remain relatively pain free. Many patients are making long diagnostic and therapeutic odysseys due to relentless pain of unclear etiology. Although numerous risk factors for chronic pain have been found, there is no straightforward common answer to the above questions of persistent pain.

Chronic pain, i.e., pain that lasts more than 3 months (1), is thought to be principally due to central sensitization (2–4), which is driven by neuroinflammation in many chronic pain conditions (5–7). Nevertheless, the mechanism of transition of acute to chronic pain remains abstract despite arduous research (8–15) and amounting data on relevant molecular and physiologic factors. In terms of the evolutionary purpose of pain, the answer to the question why acute pain transforms into chronic pain is simple: because tissue damage, i.e., noxious input and resultant nociceptive stimulus (16), persists. While the importance of initial noxious trigger in the development of sensitization and chronic pain has been well recognized, the current theories of chronic pain tend to be focused on susceptibility to and central processing of pain (8, 17–19). A clinical category of nociplastic pain has been recently introduced to cover persistent pain supposedly driven by central sensitization without any peripheral input (20). However, experimental research that supports autonomous (continuing input-independent) central sensitization is relatively scanty (4, 5) and is outweighed by contrary evidence (21–24). Although central mechanisms of sensitization are important, they cannot completely account for such chronic pain features as ongoingness, spontaneity, and, most importantly, individual variability in occurrence. If central processes, such as, e.g., synaptic potentiation and disinhibition (5, 16, 25, 26), would invariably persist on their own after disappearance of noxious trigger, then chronic pain would likely afflict all patients with identical chronic pain-prone conditions.

However, where can nociceptive input come from when there is no obvious pathology? Could the cause be an occult tissue lesion generated by the initial noxious input? Compressive nerve lesions often arise in this way, specifically, because of changes of paraneural tissue. Peripheral nerve injury as one of the causes of chronic pain has been well acknowledged (5) but primarily linked to neuropathic pain (27–29). Yet, there is increasing evidence of neuropathic etiology of chronic pain in conditions commonly thought to be of non-neural origin, e.g., complex regional pain syndrome (CRPS) type I (30–33), fibromyalgia (34–36), and a number of other diseases found to be associated with small fiber neuropathy (SFN) (37–39). The unitary neural etiology of chronic pain is becoming particularly evident from the high prevalence of accompanying SFN, the causes of which involve peripheral neural damage of various origin (40, 41), including compressive neuropathies (42, 43). Signs of peripheral neural damage after *non-neural* tissue injury have also been observed in animal experiments (44–46). The peripheral nerves are very susceptible to overload: nerve function can be disturbed by pressures as low as 30 millimeters of mercury (47), and experimental behavioral neuropathy along with ongoing neuronal activity develops within three weeks of repetitive overuse (48, 49). The most prevalent anatomic sites of chronic pain include the extremities and lower back (50–54), which are mobile and subject to overload areas, tightly interconnected with the spinal plexuses. Endoneurial edema, which can cause nerve conduction block, may quickly develop because of repetitive compression (55) at these sites. Notably, it has been hypothesized that proximal neural tissue irritation by repetitive activities may be an ectopic cause of repetitive strain injury (56, 57) (also known as nonspecific arm pain).

This report (for synopsis see the Supplementary File “Overview”) introduces a working hypothesis to explore whether sensitization maintained by peripheral proximal neural lesion (PNL), specifically by compressive PNL (cPNL), could explain all types of general chronic pain. The following sections involve hypothesis-driven reasoning, which is supported by the literature concerning sensitization, chronic pain, and neural lesion, with particular reference to PNL. Citation preference was given to the most recent review studies and original research that includes reviews and/or discussions of the pertinent issues. However, historical precedence of research was also considered if directly applicable to the central concept of the current article. The view that sensitization and chronic pain can be maintained without *persistent* peripheral noxious input (4, 20, 58) is a key point of controversy addressed by the current article.

## Pre-hypothetical reasoning: ectopic nociceptive sensitization and definition of proximal neural lesion

### General remarks on sensitization after nerve injury

Peripheral nerve lesion, including nerve compression, as a trigger of peripheral and consequent central sensitization [i.e., hyperexcitability and resultant hyperactivity of peripheral and central sensory pathway, respectively (5, 59)], has been documented by numerous studies (25, 27, 60–65). Nociceptive sensitization causes tissue hypersensitivity: hyperalgesia to noxious stimuli and painful sensations (allodynia) to non-noxious stimuli (16). Transient local tissue hypersensitivity is a common experience after traumas and in inflammatory conditions that involve skin. Clinically important central sensitization manifests as abnormal extraterritorial hypersensitivity of non-lesioned tissue and seems to be an *inevitable* result of peripheral sensitization [see, e.g., Satkeviciute and Dilley (66) and Torebjörk et al. (67)], which in turn can be induced by tissue injury, inflammation, and nerve injury (25, 68). Notably, nerve damage produces stronger sensitizing input than peripheral non-neural lesions: skin hypersensitivity lasts up to 24 h after capsaicin injection (69), ten days after incision (70), and over two months after nerve injury (65). Sensitizing effects of nerve injury obviously also apply to the nociceptive *nervi nervorum*, activity of which may account for peripheral nerve trunk pain (71–74). Clinically, sensitization-induced pain hypersensitivity can be measured as reduced pressure pain threshold (PPT) (75).

Symptomatic nociceptive sensitization can be induced by both distal neural lesions (63, 76) and proximal ones (63, 77) (as well as by non-neural insults). Why then should proximal neural injury be so important?

### Ectopic ongoing (spontaneous) nociceptive neuronal activity and neuroinflammation

Hyperexcitability (i.e., sensitization) and *consequent* hyperactivity of sensory neurons enables continuing generation and discharge of neural impulses, which is necessary to be processed by the brain cortex as persistent pain. Increased excitability of sensory neurons is due to reduction of activation threshold of their ion channels, which may be caused by nerve injury, inflammation (arising from both neural and non-neural tissue), and by ion channel mutations (68, 78, 79). Although there are many mechanisms involved in sensitization, abnormal peripheral neuronal activity plays a key role as a triggering input (25, 26, 80). In this regard, nociceptive sensitization can electrophysiologically be understood as ongoing (spontaneous) activity and mechanical sensitivity of primary sensory fibers (81), which relates to neuronal hyperexcitability. The terms of neuronal ongoing activity and spontaneous activity have largely been used interchangeably (or rather as each other complementing terms): it is generally meant that ongoing generation and discharge of neuronal impulses occurs spontaneously. Ongoing activity can be induced with varying predominance in all types of sensory neurons (82, 83) and seems to be in part driven by neuroinflammatory mediator and cytokine mechanisms regardless of the triggering insult type (5, 59). Sensitization of nociceptive neurons involves complex molecular/ionic signaling cascades as well as activation of multiple receptors and ion channels. In-depth reviews of the nerve damage-related molecular mechanisms of nociceptor sensitization are available elsewhere (16, 68, 84, 85).

Neural impulses (action potentials) can be generated not only normally by stimulation of receptors in end organs (e.g., in the skin), but also ectopically by directly stimulating sensory nerve fibers (68, 85) as well as neuron cell bodies (86), which occurs in nerve injury and radiculopathy, respectively. After peripheral nerve damage, which causes loss of some dorsal root ganglion (DRG) neurons (62, 87–89), spontaneous ectopic activity arises in the surviving afferent neurons. Obviously, ectopic nerve trunk stimulation induces activity of the entire sensory neuron *via* normal orthodromic activation of the somata of the dorsal root ganglion neuron (DRGn) and *antidromic* (retrograde) nerve ending activation (81, 90).

It has been well established that hyperexcitability of peripheral sensory neurons is an ectopic cause of central sensitization and neuropathic pain (59, 60, 83, 91–94). This also applies to neurons of the trigeminal ganglion (60, 95) and, by analogy, possibly to sensory ganglions of other cranial nerves. Ectopic discharge (i.e., arising not from nerve ending receptors) can come both from the afferent fibers (25, 29, 86, 96–99) and from the somata of the DRGn (86). Recently, however, it has been discovered that spontaneous sensory activity in a chronic nerve constriction model is generated by a structure known as the axon initial segment that is located in the prebifurcation (stem) part of the sensory fibers within the DRG (100). Ectopic peripheral neural activity is explainable by nerve injury-induced DRG inflammation and consequent activation of glial cells (5, 59, 101, 102), which leads to sensory neuron hyperexcitability (5, 59, 102). Importantly, both ongoing ectopic activity and mechanical sensitivity of afferent fibers (both of the A- and C-type) can be induced not only with but also without structural nerve injury, specifically, by focal neuroinflammation (81, 103–106) that may presumably be produced by injury of paraneural tissues (82, 107). However, while axonal mechanical sensitivity can be induced by *atraumatic* non-inflammatory axonal transport disruption (73, 82, 107, 108), ongoing activity does not develop without focal neuritis (82, 107). In reality, though, disruption of axonal transport does not seem to occur without concomitant neuroinflammation, because signs of focal inflammatory reaction are evident within hours after nerve compression (47). Another explanation of nerve injury-induced sensitization of intact nociceptors is Wallerian myelinated fiber degeneration (98) and, specifically, its neuroinflammatory effects (109). Local peripheral neuroinflammation obviously results in DRG neuron body sensitization, because neuritis-induced ongoing nociceptive fiber activity can be recorded both distally and proximally to the DRG (81).

Sensitized primary sensory neurons may cause ectopic activity of central nervous system (CNS) neurons and thus induce central sensitization (5, 21, 25), possibly *via neurogenic* CNS neuroinflammation that may presumably be generated by peripheral sensory neuron activity (110). Central sensitization can also be caused by direct CNS damage, which may lead to secondary peripheral sensitization by retrograde excitation of DRG neurons (5, 111). CNS involvement can account for bilateral neuropathy symptoms, which may be due to DRGn hyperactivity-induced hyperexcitability of wide dynamic range neurons and activation of commissural neurons of the spinal cord (66), or, also supposedly, due to systemic neuroinflammation-mediated mechanisms (42, 66). Extraterritorial hypersensitivity may be shaped by neuron crosstalk not only in the CNS (112, 113) but also in the DRG (83). Notably, allodynia involves functional interplay between nociceptive and non-nociceptive pathways (2, 16).

### Intra- and para-neural fibrosis

The above reasoning allows formulating the question of transition to chronic pain as follows: Why should DRGn hyperexcitability persist after the causative lesion has long healed?

A permanent consequence of healing of damaged tissue is fibrosis. The strength of fibrous tissue reaches its maximum at about three months after cutaneous injury (114), which, along with accompanying avascularity and acellularity of the scar, suggests that resolution of fibrosis that continues beyond three months becomes unlikely. Probably not coincidentally, this accords well with the time point given in the definition of chronic pain (1). As a part of reparative process following injury, fibrosis occurs both in the CNS (115, 116) and in the peripheral nervous system (116, 117). Signs of intraneural and extraneural fibrosis can be seen as early as three weeks following experimental repetitive overuse injury (48). Intralesional or paraneural postinjury fibrosis can presumably cause persistent focal nerve compression (56) and consequent neuroinflammation that leads to DRGn hyperexcitability. Intraganglional fibrosis likely follows direct DRG injury, e.g., due to intervertebral disc herniation. A small study found DRG fibrosis only in persistent post-herpetic neuralgia cases but no DRG scarring in pain free patients (118). Two studies have reported ectopic (i.e., remote to the injury site) fibrosis of the DRG after nerve compression (119, 120). Nerve injury-induced intraganglional inflammation (59, 101, 102), DRGn death, and resultant elimination of cell debris *via* inflammatory processes may account for ectopic fibrosis of the DRG.

### Intermittent (dynamic) partial compressive nerve lesion as an essential cause of persistent sensitization and pain

There are certain controversies in the above discussion of ectopic neural fibrosis. Natural cell death (apoptosis) occurs in almost all tissues without notable residual fibrosis and consequent functional deficits. Another doubt is whether ectopic postinflammatory *structural* neural changes *alone* could be sufficient to maintain DRG and consequent CNS sensory neuron hyperexcitability (i.e., chronic pain), because fibrotic tissue is physiologically silent. The excitatory effects of paraneural or intraneural fibrosis may fade out after eventual death of the involved neurons and resolution of associated inflammation. Importantly, experimental research has shown that *sensitization effects resolve without continuing focal neuroinflammatory input* (82, 121) (which implies existence of occult peripheral neural damage in unexplained chronic pain). This can be supported by, e.g., a carpal tunnel syndrome (CTS) study that indicates that an extreme degree of distal nerve compression, in contrast to that of a moderate grade, does not result in peripheral tissue hypersensitivity (122). The latter may be explained by complete resolution of the distal focal inflammation and its ectopic effects due to significant loss of the neural tissue. Some pain-related neuropeptides have been found to be more expressed after experimental partial nerve injury than after complete neurotomy (123, 124). Also, inflammatory changes in the DRG and spinal cord tend to be more pronounced after nerve chronic constriction than after complete transection (125). Therefore, persistent *intermittent* (*dynamic*) *partial* proximal peripheral nerve damage (*via*, e.g., external compression by myofascial tension) with resultant continuing focal neuroinflammation and consequent hyperexcitability of the DRGn could be a reasonable explanation of the above controversy.

However, although established fibrosis is physiologically silent, scarred paraneural tissue can cause intermittent neural damage by restricting mobility of nerves (48). Via this mechanism, intraneural fibrosis of peripheral nerves may also result in dynamic intraneural compression of intact nerve fibers and their bundles. These structures are relatively mobile: loose connective tissue allows movement of nerve fibers and fascicles in respect to each other (121, 126), which may lead to their internal entrapment in epiperineural scars during body motion. Thus, motion of the healthy part of the nerve may result in dynamic compression even in seemingly static neural scarring. Although such a subtle mechanism of dynamic intraneural nerve compression may probably produce only slight disturbance of nociceptive neuron function, this may be sufficient to maintain central sensitization *via* long-term potentiation, which involves augmentation of action potential output by recruitment of subthreshold inputs (25).

Furthermore, nerve compression may intermittently be produced not only by neuromuscular but also by neurovascular mechanisms, e.g., as in trigeminal neuralgia (127). The neurovascular mechanism (presumably involving reflexive blood vessel dilation or spasm and transient edema) may be responsible for both intraneural and extraneural compression. These modes of intermittent neural compression may act concurrently. In naturally immobile areas like the skull, dynamic neural compression can probably only be produced by the neurovascular mechanism.

It is noteworthy, however, that sensory nerves are partly motile because they usually traverse dynamic structures or terminate in them (e.g., trigeminus branches glide because of facial muscle activity). Therefore, both para- and intra-neural fibrosis probably always result in intermittent nerve compression. Mobility of the sensory nerves may be responsible for intermittence of compression and resultant sensitization in numerous entrapment syndromes.

### The double crush concept and sensitization

As a bifocal nerve lesion, the double crush syndrome (128, 129) may act as a strong trigger of sensitization. As noted above nerve compression physically impairs intra-axonal circulation and induces focal neuroinflammation (47). Disruption of axoplasmic transport in turn causes axonal mechanical sensitivity (73, 82, 107, 108), while neuroinflammation induces both axonal mechanical sensitivity and ongoing nociceptor activity (103, 104). In double crush syndrome, secondary nerve compression is facilitated by impairment of axoplasmic transport due to primary entrapment (128, 130). The resultant neurological deficit has been found to be greater than the sum of the deficits produced by each of the lesions individually (131, 132). Therefore, theoretically, simultaneous action of two or more nerve lesions, which would be asymptomatic if separately applied, may produce clinical nerve injury (133) by synergy of their effects and eventual activation of the DRGn (Supplementary Figure 1S). Similarly, compressive nerve lesions and coexistent *systemic* neuroinflammation may produce a cumulative effect, because neuroinflammation also disrupts axoplasmic transport (108, 134) (probably because of compression consequent to inflammatory neural edema). On the other hand, nerve compression induces focal neuroinflammation on its own (47, 135), which is essential in development of ongoing activity of nociceptive neurons (82, 107). Systemic inflammatory non-neural tissue conditions may further contribute to sensory neuron hyperexcitability (78), possibly, *via* induction of neuroinflammation (5). Thus, in patients with background systemic neuroinflammation, a subclinical focal peripheral nerve injury may lead to sensitization or its enhancement (Supplementary Figure 2S). Also, peripheral nerves may become hyperexcitable *via* other systemic factors (128), including genetic mechanisms, as has been shown with mutation of Na(v)1.7 sodium channels in SFN (136, 137) (however, there is no congenital pain).

### Distal vs. proximal neural lesion

As discussed above, distal peripheral nerve lesions inevitably lead to proximal neural involvement in the form of the DRGn ectopic hyperexcitability and, possibly, DRG fibrosis. Furthermore, peripheral nerve injury results not only in loss of DRG neurons [proximal injuries being more detrimental (87, 88, 138)], but also in death of neurons in the dorsal horns of the spinal cord (62). Therefore, one can presume that distal nerve damage eventually generates a secondary (ectopic) PNL manifesting as structural and/or functional changes in the DRG and CNS. Logically, in such cases both distal nerve lesion and the ectopic PNL would coexist and shape the clinical picture of neuropathy.

The above elaboration on the ectopic consequences of distal peripheral nerve damage indirectly attests to the significance of direct proximal-level nerve injury. The closer the injury to the DRG, the more neurons are lost (87, 88, 138) and the greater overall excitation of DRG neurons is effected, because obviously more fibers are damaged in proximal nerve injuries than in distal ones. Similarly, Wallerian myelinated fiber degeneration, which induces spontaneous activity of the neighboring uninjured C-fibers (98, 109) and thus contributes to sensitization, involves a longer length of the nerve distal to the damage (and consequently can produce greater cumulative sensitizing effect) in proximal than in distal nerve lesions. Proximity of neural lesion to the DRG may also be important in that spontaneous neuron activity originates in the axon initial segment located in the DRG (83, 100).

Widespread nociceptive hypersensitivity, a sign of central sensitization, often accompanies carpal tunnel syndrome (71, 122, 139, 140). However, widespread hypersensitivity has been found absent in CTS patients without neck and arm pain (141), which implies that concomitant cPNL (a likely cause of the pain) may have been absent in those patients. This further implies that proximal-level neural damage may play a more significant role in the development of sensitization than distal peripheral nerve lesions alone. Widespread hypersensitivity in cervical radiculopathy (142), lumbar radiculopathy (143), whiplash injuries (142, 144), and plexopathies after breast cancer surgery and radiotherapy (145) also suggests the etiological importance of proximal compressive nerve injury. Of similar relevance may also be the occurrence of bilateral peripheral hypersensitivity in patients with non-neurogenic neck pain (146) and nonspecific arm pain (147).

### A summarized definition of PNL, functional PNL, and compressive PNL

Summarizing the current section, and for the purpose of this article, PNL can be defined as a primary neural lesion in the proximity of spinal plexuses, sensory ganglia, and/or in the CNS, as well as a secondary (ectopic) ganglionic and/or CNS sensory neuron lesion induced by indirect injury. Also for the purpose of this paper, the functional PNL is understood as sensory neuron hyperexcitability and its consequent hyperactivity. Neuron hyperexcitability results from focal neuroinflammation, which in turn can be generated by proximal dynamic (intermittent) paraneural and intraneural compression, i.e., *via* peripheral cPNL. In this report, PNL primarily refers to peripheral lesions.

## A working hypothesis of chronic pain maintained by dynamic compressive PNL

On the basis of the above considerations, it can be hypothesized that general chronic pain and pain in peripheral tissue conditions that are usually painless is due to nociceptive sensitization produced by hyperexcitability of sensory neurons (i.e., by functional PNL), which in turn is induced and/or maintained by persistent dynamic peripheral cPNL. For simplicity, this postulation will be elucidated below by initially addressing unusual local pain and then discussing chronic pain in general. This partition is only conditional because both pain categories refer to persistent pain driven by the same mechanism.

## Compressive neuropathic etiology of pain in usually painless conditions

### Unusual pain in space occupying lesions

**Figure 1:**
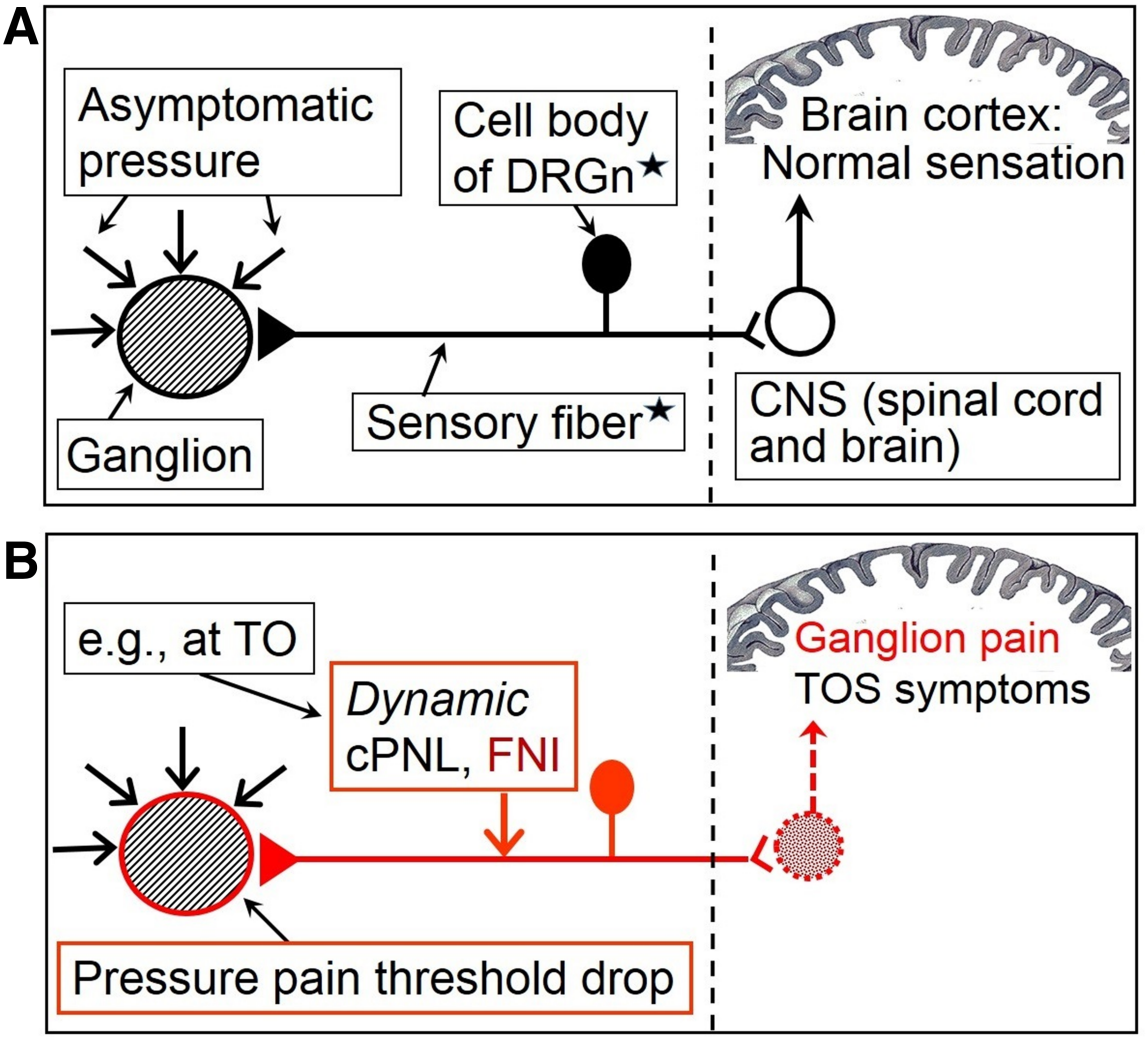
Hypothetical simplified mechanism of isolated pain in the case of a usually painless ganglion cyst. (**A**) Painless ganglion in the absence of sensitization. (**B**) Sensitization induced by compressive proximal neural lesion (cPNL), which is here clinically presented as TOS, induces focal neuroinflammation and consequent reduction of excitation threshold of afferent neurons. This results in PPT decrease of peripheral tissue and ganglion pain. This figure can serve as an explanation of neuroma pain as well; the ganglion then would represent a nerve stump including receptors of sensitized *nervi nervorum* (represented here by the DRGn). Filled in triangle = receptor of the sensory neuron; red arrow = force that is sufficient to produce symptomatic compression of otherwise unaffected nerve; black triangle with black horizontal line and black DRGn = peripheral nociceptive pathway in a normal state; red triangle with red horizontal line and red DRGn = hyperexcited peripheral nociceptive pathway; white circle = neuron of the dorsal horn of the spinal cord (arrow shows impulse direction); red pattern-filled circle = hyperactive dorsal horn neuron (arrow shows impulse direction). DRGn, dorsal root ganglion neuron; CNS, central nervous system; TO, thoracic outlet; cPNL, compressive proximal neural lesion; FNI, focal neuroinflammation; TOS, thoracic outlet syndrome. *Diagrammatically represents a mixture of primary sensory neurons (both of A- and C-type), functional interplay of which is involved in producing complex neural symptomatics. The complex involvement of central sensitization and neuron crosstalk, responsible for mechanical hyperalgesia and allodynia, is not shown. For more comprehensive depiction of nociceptive pathways and processes, see figures in the supplement file “Overview”.

Space occupying lesions generate intralesional pressure, exert certain pressure on the surrounding tissue, and are equally compressed by the latter, which in areas of nociceptive hypersensitivity can cause local pain because of pressure pain threshold drop and consequent mechanical allodynia and hyperalgesia (Figure 1) that commonly accompany sensitization (16, 75). Therefore, cPNL-induced and/or -maintained sensitization may be the cause of pain in usually painless surgical conditions, e.g., ganglion cyst (148), Dupuytren's fibromatosis (149), and scars (150). Obviously, such lesions can also be painful to palpation during clinical examination when background hypersensitivity coexists. Differently from general chronic pain (see below), persistent pain in local lesions may be caused not only by paraspinal cPNL but also by compression at any distal level *proximal to the lesion*. This concept of the hypothesis finds an indirect confirmation in a clinical study in which oral cancer patients with perineural invasion showed greater pain scores than patients without nerve involvement (151). The latter study also suggests that proximal peripheral nerve injury-induced sensitization can augment preexistent organic pain.

### Compressive PNL as an etiology of neuroma pain

There are no clear histological criteria to distinguish between painful and painless neuroma (152). Therefore, painful neuroma as a local condition with unexplained pain (153, 154) can be taken as a special case of application of the cPNL hypothesis. Logically, as a chronic pain condition, painful neuroma should have a source of nociceptive sensitization. Compressive forces naturally occur in and around neuroma itself because of the space-occupying nature of this lesion and fibrosis. Consequently, neuroma pain may be a result of cPNL-induced and/or -maintained sensitization of nociceptive *nervi nervorum* of the nerve stump (analogously as shown in Figure 1) and, importantly, a consequence of axonal mechanosensitivity that may also be induced and/or maintained by cPNL. This concept is based on the following neurophysiological logic, which is in a close context to the above prehypothetical reasoning.

A typical manifestation of symptomatic neuroma includes the Hoffmann-Tinel sign (155) that reflects nerve trunk mechanical sensitivity (107). Mechanical load of ectopically excited intact nociceptive nerve fibers is thought to produce neuropathy symptoms like radiating pain (82, 103, 105, 108), which is also characteristic of painful neuroma. In this regard, it is known that mechanical excitability of nociceptive axons can be induced by direct axonal injury [e.g., compression (156) or transection (97, 99)] and by focal neuroinflammation (73, 103, 105, 121), which is likely to accompany all nerve injuries (101). On the other hand, axonal mechanical sensitivity *disappears with fading of the noxious input*, as has been shown in an experimental model of non-inflammatory axonal transport disruption (82, 107). From a neuritis model, the same could be said about both mechanosensitivity (103, 105, 121) and spontaneous ongoing nociceptive fiber activity (82). [In these models, dissipation of noxious stimulus activity resulted in disappearance of cutaneous hypersensitivity as well (66, 105, 157, 158).] Increased abnormal afferent activity of transected peripheral nerve fibers continually decreases after the third postinjury week (97).

There is no active neuroinflammation or other pathophysiological activity within healed nerve stumps, which could contribute to ongoing sensory neuronal activity. Consequently, neuroma alone as a healed nerve injury cannot act as a permanent noxious stimulus and, therefore, cannot maintain clinically manifest sensitization accompanied by axonal mechanosensitivity (otherwise, all neuromas would probably be painful). Coexistence of neuroma and of cPNL-induced localized neuroinflammation (i.e., of a sensitizing input) may be crucial in the pathophysiology of neuroma pain. This logic practically explains why only some neuromas are painful: not all neuroma patients have significant background cPNL that induces and/or maintains nerve trunk hypersensitivity. Notably, nociceptive *nervi nervorum* can be sensitized by mechanical and chemical stress (74, 159), factors which may occur in nerve compression and consequent focal neuroinflammation.

Proximal neural lesion can be well expected (but may easily be overlooked, as discussed below) in painful neuroma that is often a result of a nerve injury sustained during trauma, because traumas can result in cPNL that clinically may present as, e.g., thoracic outlet syndrome (TOS), traction plexopathy with neural fibrosis, and cervical or lumbar radiculopathy. Likewise, coincidence of neuroma and pre-existing non-traumatic radiculopathy or TOS may also result in neuroma pain. Notably, painful neuromas have been mentioned to accompany the double crush syndrome (153). Also of relevance, the presence of concomitant cPNL may explain why surgical treatment of painful neuroma mostly produces only partial improvement (160, 161).

Intermittent nature of nerve compression may be more important in maintaining sensitization than paraneural or intraneural fibrosis as such. Therefore, indirect support of the above neuroma concept could also be the rarity of painful traumatic neuromas in areas innervated by naturally less mobile nerves, e.g., by the trigeminus (162) and other cranial nerves, because these nerves are less likely to be affected by overuse and intermittent cPNL. Probably, because of the same reason painful neuromas occur relatively rarely in the head (163). Of note, while head skin lacerations are common but rarely result in painful neuromas, these occur relatively often in extremities (154) that are extremely mobile. Furthermore, the rare symptomatic neuromas of the oral cavity occur predominantly in the distribution of the mandibular nerve (164), the most mobile division of the trigeminal nerve (see also the relevant discussion below).

Increased accumulation of ion channels has been observed in painful neuromas (165–167). This may also be due to effects of cPNL, because nerve compression is accompanied by expression of novel ion channels (135). Furthermore, migration of ion channels has been observed in an experimental nerve constriction injury (168).

## Compressive neuropathic etiology of chronic pain in general: vicious cycle of chronic pain

Chronic pain may be caused by compensatory overuse-related cPNL. This can be judged, e.g., from the occurrence of painful musculoskeletal conditions in amputees (169–171). Initial posttraumatic pain may result in postural protection of the painful area and consequent compensatory musculoskeletal overuse. This may start a cycle of muscle weakening of the involved area and compensatory overuse of uninvolved muscles with resultant damage of the both muscle groups. Weakened muscles are naturally more susceptible to overuse injury. Muscle weakness and compensatory imbalance may cause myofascial tension, which can result in compression of proximal nerves in many systemic and posttraumatic conditions. This mechanism may explain high prevalence of chronic pain in patients with disabilities (172, 173). Notably, risk factors for fibromyalgia such as older age, high body mass index, and pre-existing medical disorders (174) could be associated with risk for muscle weakness. On the other hand, there may be reciprocity between loss of muscle strength and cPNL, because the latter is often accompanied by motor fiber damage. Wrong posture, ligament laxity, and muscle shortening may also contribute to development of cPNL, as has been implied by a biomechanical explanation of TOS pathogenesis (175). Another TOS study has suggested that abnormal postures due to initial nerve compression may lead to neck muscle tightness, imbalance, compensatory overuse, and consequent secondary worsening of the neural entrapment (133). Intermittent nerve compression *via* muscle spasm, which is common in proximal neural lesions, e.g., radiculopathy (176), may play an important role in this cycle. It is also of note that, as earlier suggested, nerve irritation by injured paraneural tissue may maintain focal neuroinflammation, which can serve as a persistent noxious input that causes nociceptive fiber hyperexcitability (82, 107).

**Figure 2:**
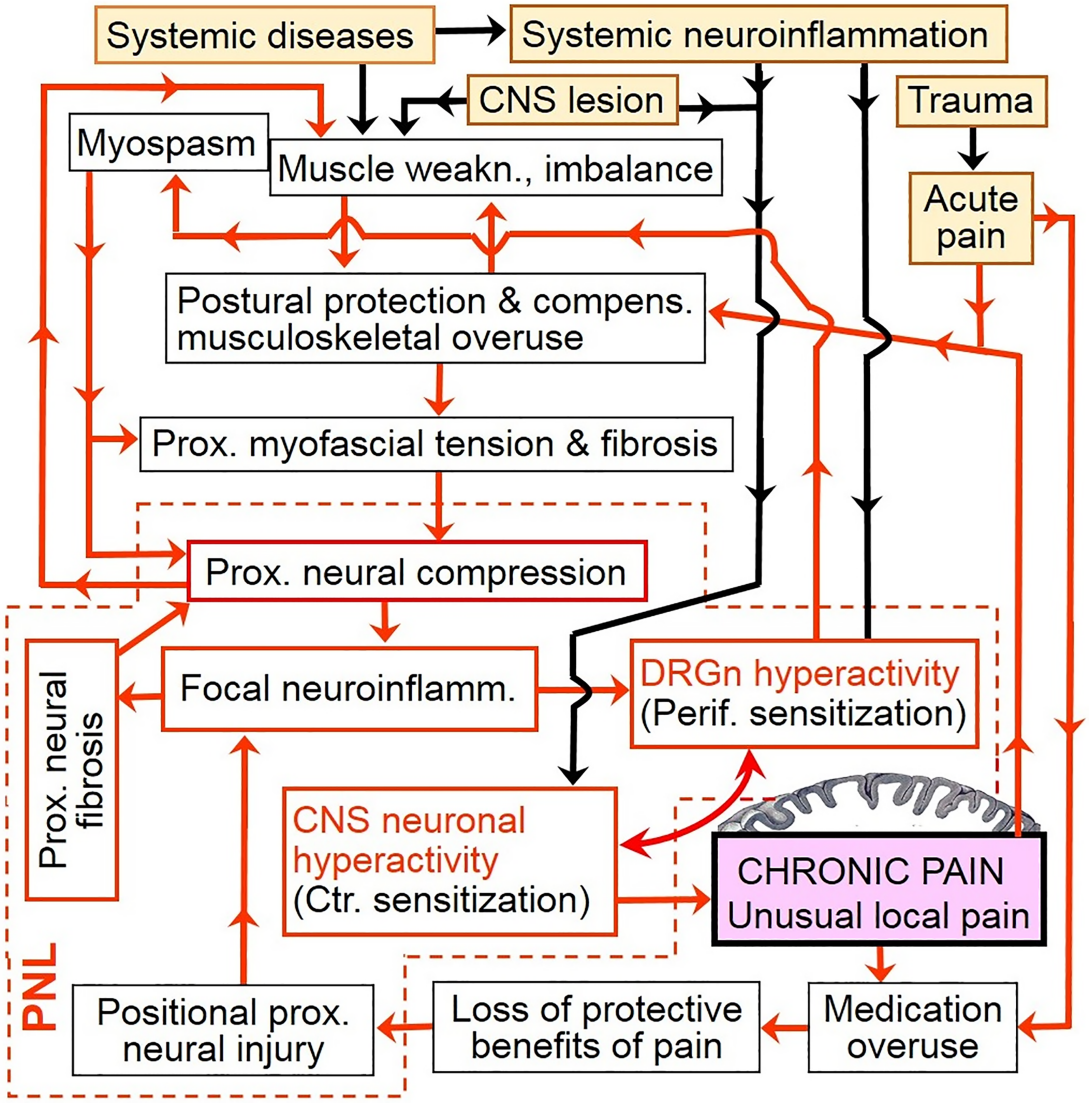
Chronic pain as a vicious cycle of persistent aggravation of proximal neural lesion. Only general etiological events (yellow filled-in boxes) are shown. Note the key role of focal neuroinflammation and DRGn hyperexcitability (i.e., functional PNL). Because of cPNL-maintained focal neural inflammation, ectopic firing from hyperactive DRG neurons can continue and evoke pain even after the initial etiological event has healed. This figure includes several cycles that can be discovered by following any arrow until the text box “Chronic pain” is reached; from there any arrow would eventually lead back to the latter textbox. (See also simplified versions of this illustration in Figure 3 and in Overview Figure 5ov in the Supplementary File “Overview”). Yellow filled-in text boxes = initial etiological events; redlined boxes = major drivers of the vicious cycle; violet filled-in box = processing of nociceptive impulses by the brain cortex; black flow arrow = triggering input which is not permanently involved in the vicious cycle; red flow one-headed arrow = input which serves as a link of the vicious cycle; bowed double-headed arrow = reciprocal enhancement but *not an independent cycle*. weakn., weakness; compens., compensatory; Prox., Proximal; neuroinflamm., neuroinflammation; DRGn, dorsal root ganglion neuron; Perif., Peripheral; CNS, central nervous system; Ctr., Central; PNL, proximal neural lesion.

**Figure 3:**
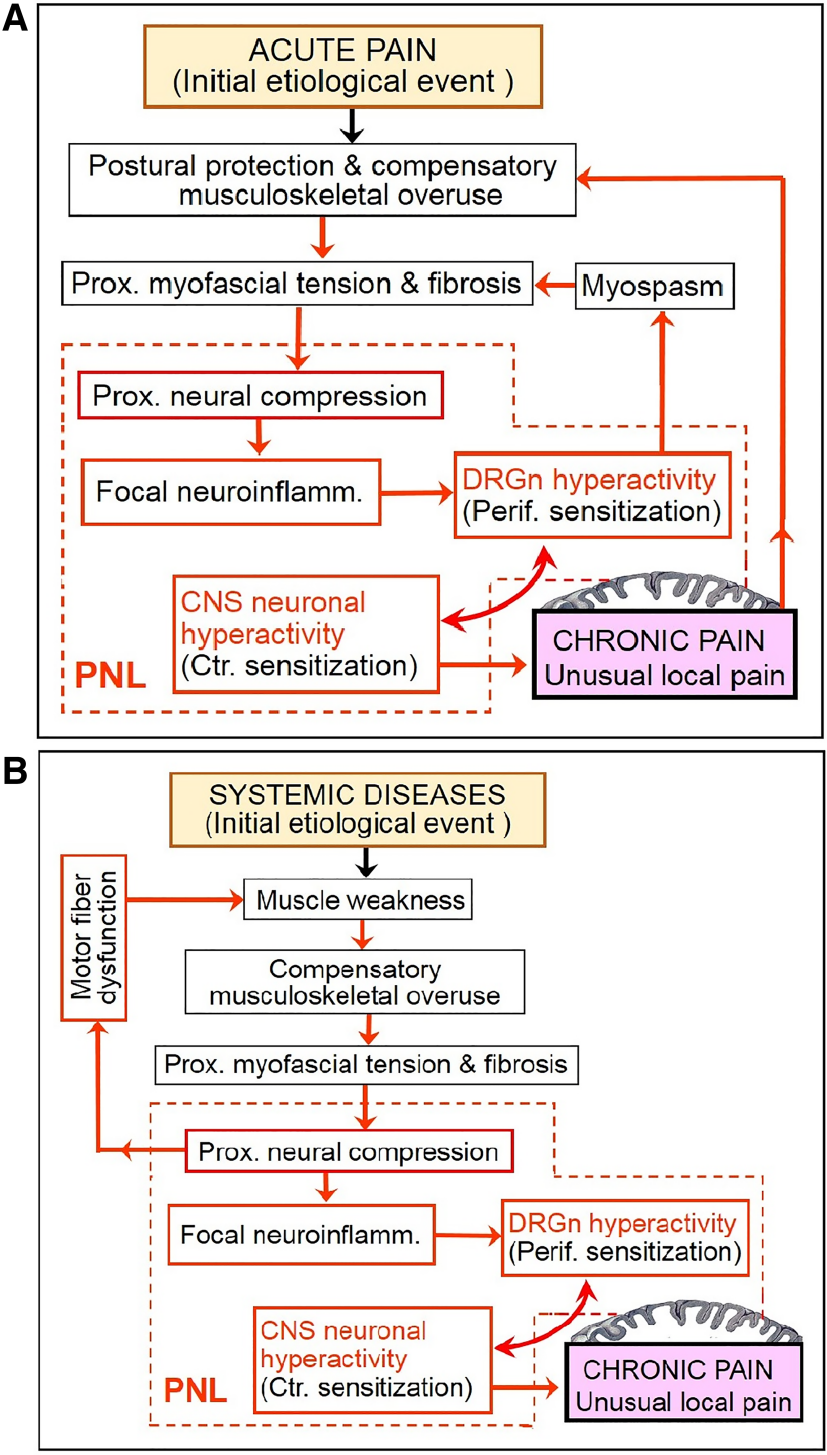
Partial deconstruction of the vicious cycle shown in Figure 2. (**A**) Two subcycles initiated by acute pain. (**B**) A subcycle initiated by systemic musculoskeletal impairment reveals the role of cPNL-induced motor fiber dysfunction (which is not shown in Figure 2). See Figure 2 legend for explanation of abbreviations and pictograms.

Considering the above, chronic pain may be a consequence of a vicious cycle that involves continuing aggravation of PNL. This cycle may start with hyperexcitability of DRG neurons due to, e.g., peripheral tissue damage or radiculopathy, then proceed to consequent proximal reflexive muscle spasm and myofascial tightness, and eventually produce (or aggravate) focal neuroinflammatory injury of the proximal nerve trunk due to its compression at anatomically narrow spaces. In other words, functional PNL (i.e., DRGn hyperexcitability) may induce cPNL and may be maintained by the latter *via* focal neuroinflammation at the compression site. The reciprocity between DRGn hyperexcitability and cPNL can drive the vicious cycle even when the initial etiological trigger has healed (Figures 2, 3A). Importantly, the cycle may be perpetuated by reciprocity between cPNL-caused motor fiber dysfunction and muscle weakness that leads to muscle imbalance and compensatory overuse (Figure 3B). Primary CNS lesions may ignite the vicious cycle *via* possible antidromic activation of the DRGn (5, 111). Sensitization may play a role of a catalyst of this cycle, because sensitized nerve trunks become more susceptible to further damage (see discussion of the double crush concept and neuroma pain above). Thus, transition of acute to chronic pain may be due to the negative postural and compensatory overuse-related impact of acute pain (Figures 2, 3A). Because of the latter two factors and sensitization-catalyzed nerve vulnerability, a single-site cPNL may develop into a multiple-site cPNL, which could be another explanation of bilateral widespread chronic pain. It is essential that this cycle cannot function without persistent proximal neural lesion of intermittent character, which is discussed in the prehypothetical reasoning section above.

Disorders of the CNS can also cause muscle weakness, imbalance, compensatory overuse, and resultant cPNL. However, CNS lesions, as well as distal nerve lesions, cannot be maintained and aggravated by the above neuromuscular cyclic mechanism, hence again the importance of the intermittent compressive PNL.

## Application of the cPNL hypothesis to chronic pain in general

### Why does occurrence of chronic pain vary from patient to patient?

The vicious cycle of chronic pain suggests that occurrence of cPNL, and thus of sensitizing input, may vary as vary postural muscular consequences of traumas and diseases among patients. Susceptibility to pain may in fact represent a predisposition for cPNL. This may explain why chronic pain incidence increases with age (177, 178), i.e., with gradual decline of muscle strength. Another strong epidemiologic tendency is a high prevalence of chronic pain among women (177, 179), which may be due to the relatively high susceptibility of their musculoskeletal system to overload injury (180) and thus to consequent cPNL. Genetic, neurophysiological, and other factors may play an additional role in the development of cPNL.

### Why does pain predicts pain?

Having in mind the possibility of background compressive PNL may help to explain unclear aggravation of pain in many clinical situations. Examples could be the reports that have shown that preoperative regional pain correlates with acute pain after repeat surgical deliveries in women with painful post-Caesarean scars (181) and predicts chronic pain in limb amputees (182) as well as in patients after breast cancer surgery (183, 184); pre-operative/pre-amputation pain is also a risk factor for phantom limb pain (185). In the context of the current hypothesis, one could speculate that surgical trauma may have worsened a coexistent cPNL (e.g., plexopathy or radiculopathy that are likely in the above clinical situations) and its sensitizing effects. It is also considerable that risk factors for chronic pain include trauma (186–190) and surgery (187, 190), in particular extensive surgeries (191) that may be associated with a higher risk of positional nerve injury (192). Furthermore, according to the double crush concept, background peripheral nerve damage makes the involved nerves more susceptible to further positional injury during surgery (for analogy see Supplementary Figure 2S). Noteworthy is also that trauma and surgery may share common pathogenesis of chronic pain and its risk factors, such as acute pain intensity, preinjury pain, and level of disability (190), which may be due to coincidental cPNL. Hypothetically, psychology-related predictors may be associated with occult cPNL as well. For example, familial pain may be associated with certain musculoskeletal predispositions for cPNL. On the other hand, cPNL-induced chronic pain may cause psychological disorders, the negative musculoskeletal-postural impact of which (193) may in turn aggravate cPNL.

### Explanation of chronic pain in spinal cord injury (SCI)

While hyperexcitability of both central and peripheral nociceptive neurons has been shown in SCI (DRG neurons being excited antidromically) (111), it remains unaccounted for why not all spinal cord injuries result in chronic pain [see, e.g., Hunt et al. (194)] and, specifically, why some SCIs result in “only at-level” or “only below-level” chronic pain (195). Furthermore, why does below-level SCI pain tend to occur more often than above-level pain (196)?

These conundrums could be explained by variability in occurrence of *symptomatic* cPNL above, at, and below SCI level. Chronic pain above SCI level may be due to cPNL that is produced by compensatory overuse, as earlier discussed. At and below SCI level, cPNL probably occurs in all or most patients, since, because of denervation, SCI likely results in a secondary musculoligamentous instability of the spinal skeleton at and below the injury, which may facilitate positional cPNL in the affected paraspinal regions. In addition, traumatization of spinal nerves, due to involuntary muscle contractions that accompany SCI as well as due to postural changes performed without patient's voluntary muscle control, may contribute to cPNL at and below SCI level. However, cPNL may not always become symptomatic because of variability in severity of SCI as well as in degree of recovery of dorsal horn neuron function and spinothalamic transmission. For example, if dorsal horn neurons and their connections are relatively well functioning at the injury level but there is completely damaged spinothalamic transmission from the lower segments, then one is likely to observe “only at-level” chronic pain. Similar logic can also explain “only below-level” and unilateral SCI pain. Notably, sensory and motor preservation as a risk factor for progression of neuropathic pain in SCI patients (197) may be associated with a higher risk of cPNL due to greater potential for physical activity of those patients, because they may overload proximal nerves by performing tasks that are unnaturally overdemanding for intact muscles.

The unclear mechanism of lag in the onset of chronic pain after spinal cord injury (198, 199) is explainable by gradual development of cPNL *via* positional and compensatory overuse-related trauma. Time is also required for partial reestablishment of function of the damaged sensory pathways. Here again, healed lesions (in this case, SCI) alone cannot serve as a continuing noxious input, as discussed above. Obviously, postural traumatization of healed (fibrotic) spinal cord lesions may produce both direct triggering input (which still fits into the cPNL concept) and secondary (antidromic) hyperactivation of DRG neurons.

### Explanation of trigeminal neuralgia, temporomandibular pain, and other orofacial pain

Because of relative shortness of the trigeminal nerve, its lesion at any level can be regarded as PNL. In trigeminal neuralgia, the trigeminal nerve is compressed (most often *via* a neurovascular mechanism) proximal to its ganglion (200), while in temporomandibular disorders the preganglionic part of the nerve is involved (201, 202). Trigeminal neuralgia is a rarity in comparison to temporomandibular chronic pain (203, 204). This may be due to relative immobility of the postganglionic (prepontine) trigeminal nerve compared to its mobile preganglionic division V3 (mandibular nerve). The latter anatomofunctional feature creates predisposition for motion-maintained intermittent cPNL (discussed in the prehypothetical reasoning section) of the mandibular nerve at its exit from the skull (which is very close to the trigeminal ganglion). Notably, lesions of a single peripheral division of the trigeminus may lead to hyperexcitability of uninvolved sensory neurons because of lesion-induced inflammation in the trigeminal ganglion (201) and possibly because of excitatory effects of Wallerian degeneration. Consequently, lesions of, e.g., mandibular nerve may lead to sensitization of uninjured V1 and V2 trigeminal divisions.

The enigmatic etiology of orofacial pain can be further elucidated by applying the double crush concept. Various combinations of subclinical and/or symptomatic lesions of the trigeminus within and outside the skull may result in a variety of painful symptoms (for an analogy, see Supplementary Figures 1S, 2S). For example, only neurovascular compromise of trigeminus function within the skull may manifest as the classical trigeminal neuralgia, while combination of subclinical proximal and distal involvement of the nerve may cumulate to diffuse orofacial pain.

### Overlapping pain and cPNL

Development of overlapping pain may require many months after the onset of the first pain (205). This is in line with the introduced concept, because development of an established cPNL obviously takes time, especially when there is no initial proximal neural injury. Furthermore, the neuromuscular mechanism of chronic pain cycle suggests that occurrence of cPNL at one site may lead to another cPNL in a different body region. However, single cPNL may also cause spread of symptoms because of continuing aggravation of PNL (Figures 2 and 3) and resultant involvement of commissural neurons of the spinal cord.

For example, the overlap of the jaw/face, head, and neck pain (206) may be due to an entrapment lesion at the neck (Supplementary Figure 3S-A), which can be supported by a recent suggestion that occipital nerve compression causes unremitting head and neck pain (207). Relevantly, the beneficial effects of botulinum toxin in some cases of migraine (208) may be due to resolution of cranial muscle spasm (and thus due to nerve decompression) by botulinum. The overlap between CRPS and fibromyalgia pain (209) may also be caused by a single or multiple-site cPNL, because neuropathic features are characteristic of both CRPS (210) and fibromyalgia (36) (Supplementary Figure 3S-B).

## Implications for chronic pain management

### Why should compressive PNL go so often undiagnosed?

One may argue that cPNL has distinct diagnostic signs that can only be associated with the classical neuropathic pain. However, peripheral nerve lesions are often overlooked in practical management of general chronic pain. A compelling example is the CRPS type I that historically has not been associated with nerve injury. Despite the evidence of the latter misconception (30–33), the diagnosis of CRPS type I continues to be in clinical and academic use.

Recognition of nerve involvement at the paraspinal and plexus level may be especially problematic due to the complexity of differential diagnosis. The diversity of clinical symptoms and signs of chronic pain conditions may mask cPNL as an etiology. The intermittence of myospasm-induced cPNL may present a special difficulty. Mild focal intraneural fibrotic changes can result in permanent neurological deficits, but may be undetectable by either electrodiagnostic or imaging techniques. In fact, instrumental approaches may not reveal any specific pathologic features even when proximal neural damage is clinically obvious, e.g., as in neuralgic amyotrophy (211). A specific example of diagnostic difficulty of cPNL could be the neurogenic thoracic outlet syndrome (133, 212–215) that may present with extraterritorial symptoms, e.g., headaches (133, 212), as the only concern of the patient. Local pain may also be the only complaint revealed to the doctor in the cases of unusually painful peripheral conditions caused by cPNL. While previous traumatic events are known to be a risk factor for chronic pain (186–190), common uncomplicated traumas like falls may go unreported (216). Seemingly trivial injuries may initially result in subsymptomatic proximal paraneural tissue damage and lead to serious neurogenic deficits years later because of an additive effect of paraneural fibrosis, repeated traumas, overuse injury, and age-related neural lesion. On the other hand, local musculoskeletal consequences of extensive injuries may veil concomitant peripheral neural lesions (217).

In general, chronic pain conditions very often go unrecognized (218). One study has found that the diagnosis of chronic pain was overlooked by referring physicians in 66.7% out of 60 cases, which included peripheral nerve entrapment, radiculopathy, and TOS (219). Similar chronic pain studies revealed that 40% to 80% of patients were misdiagnosed (220). Misdiagnosis in chronic pain is a challenging, complex problem (221, 222) that may be influenced by etiological uncertainty of this disorder as well as by relevant diagnostic bias and inertia. For example, over 18% out of the surveyed 804 clinical practitioners would not examine the neck in pain conditions of the shoulder (223). Given the proximity of the latter two anatomic areas, one may expect even a higher proportion of overlooked examinations of proximal paraneural areas in patients with more distal pain. The diagnostic errors may also be due to incomplete anamnesis caused by miscommunication. It has been found that up to 59% of the patients with chronic pain think they had difficulty in conveying their symptoms (224), which is not surprising considering the clinical complexity of this condition. Gathering a cPNL-based anamnesis may improve this situation.

Referral of patients with unclear pain to the right specialist may be of decisive diagnostic importance. Unfortunately, chronic pain does not fit into any single discipline of medicine, which may be why proximal neural lesions and distal painful conditions are often regarded as areas of different specialties. For example, the thoracic outlet syndrome, which produces abundant signs and symptoms in the hand, may not belong to the scope of upper extremity surgeons, but, depending on the clinical institution, may be managed by neurosurgeons, thoracic surgeons, or vascular surgeons. In this regard, the specialized treatment of general chronic pain is even more problematic, because this condition is mostly managed by primary care providers (224, 225) who have little experience in peripheral nerve diagnostics and treatment. Obviously, primary care doctors are aware to consult a large spectrum of relevant specialists. However, should diagnostic errors occur at the specialist level, then they are likely to continue long after the patients return to the primary care. The cPNL hypothesis implies that peripheral nerve surgeon's involvement may be of key significance in chronic pain management. A review by Poppler and Mackinnon (153) seems to be one of very few sources to specify peripheral nerve surgeon in the pain team context. Neurosurgeons are often mentioned in terms of interdisciplinary management of chronic pain. However, neurosurgery is primarily understood as CNS surgery. Although encompassed by the specialty of neurosurgery, peripheral nerve surgery is a multidisciplinary field that requires anatomofunctionally related expertise in musculoskeletal and other peripheral tissue conditions (226, 227), which are within the scope of orthopedics and traumatology, plastic and reconstructive surgery, and hand surgery.

### Chronic pain treatment in the light of the cPNL hypothesis

The mainstay of chronic pain treatment remains pharmacotherapy, which is of low long-term effectiveness. Poor results of chronic pain medication (228–230) may be due to undiagnosed occult proximal neural injuries. Surgery is a straightforward approach when PNL is caused by external nerve compression. Unfortunately, in longstanding entrapment lesions surgical decompression may not bring about a complete relief because of possible intraneural fibrosis and consequent permanent neurological deficits. Invasive approaches may even worsen the clinical picture if tissue hypersensitivity remains unresolved, which may occur in the cases of multifocal nerve damage. For example, when TOS and CTS coexist, release of the latter alone may not abolish the sensitization effects of TOS and could result in painful scars (Supplementary Figure 4S). [This may explain why outcomes of surgical treatment of double crush syndrome are poorer than those of isolated distal nerve compression (231, 232).] Thus, conservative treatment may be the major or the only option that can be offered to many chronic pain patients.

How could the concept of cPNL help to improve non-invasive techniques of chronic pain treatment? The varying effectiveness of non-invasive modalities (233–235) may be due to not knowing the underlying pain mechanisms. The cPNL hypothesis enables explaining why some standard approaches work *similarly* [see, e.g., the review by Ferro Moura Franco et al. (236)] in so many *different* chronic pain conditions. Determining the exact location of proximal neural lesion may help to individualize treatment.

Certain effectiveness of anti-inflammatory medication in chronic pain (237) may be in part due to abatement of local tissue inflammation and neuritis at the site of cPNL. Most conservative treatments also involve motion therapy, which is naturally associated with reduction of daily physical load. Therapeutic motion enhances tissue blood circulation and facilitates gliding of nerves, which may have positive effects on neuropathic pain (238). Mild exercises and load reduction may also relieve myofascial tension that causes nerve compression. The effectiveness of sympatholytic treatments in the sympathetically mediated chronic pain (239) can also be explained by the cPNL hypothesis. Sympatholytic approaches enhance intraneural and muscular circulation at critical sites because of vasodilatation, which can eventually result in myofascial release and improvement of neural function. The therapeutic effects of acupuncture (240) may be due to an analogous mechanism. Changing lifestyle and employing psychological modalities like cognitive behavioral therapy have the benefit of offloading nerves and encouraging patient's self-management.

Patient's active participation in treatment may influence outcomes. For example, lack of patient control during physiotherapy may result in neural overload and thus worsen proximal nerve damage. Similarly, overuse of chronic pain medication (241) may lead to loss of the protective benefits of pain, especially during sleep, and thus aggravate nerve lesion (Figure 2). Analgesia-facilitated deep sleep is associated not only with patient's comfort, but also with an increased risk of positional nerve injury, e.g., as in the case of the “Saturday night palsy” (242) or during general anesthetic surgeries (192).

### Why does peripheral tissue surgery help?

Why then do local surgical approaches work in many cases of unusually painful isolated conditions? Surgery can bring about an improvement because of elimination of the sensitizing trigger by, e.g., freeing nerves from scars (243) or by insulating neuromas (160). However, most important may be postoperative rest, which can produce resolution of sensitization by offloading the involved proximal nerves. Furthermore, the postsurgical patients are likely to receive physiotherapy, which may have a positive effect on chronic pain due to myofascial release (244) and nerve mobilization (238). Such effects of postsurgical management may explain, e.g., why improvement after painful neuroma surgery is not clearly associated with employed surgical techniques (160, 161).

## Testing the cPNL hypothesis

As noted in the introduction, a distinct feature of the proposed hypothesis is the presupposition of persistence of noxious input *via* peripheral proximal neural lesion. In other respects, this postulation does not challenge current theories of chronic pain. Neurophysiologically, the cPNL hypothesis may be compatible with most other explanations of persistent pain. Therefore, frameworks of designs of previous relevant chronic pain studies could be adapted to testing the current hypothesis.

Experimentally, the hypothesis could be tested by exploring DRG neuron damage in association with *postural changes* in *non-neural* injury-induced pain. For example, exploration of referred hypersensitivity in experimental knee osteoarthritis in animals (245) may reveal a possible etiological influence of posture-induced cPNL. (There may be a similar relationship between the negative postural impact of osteoarthritis (246–248) and referred hypersensitivity in human knee osteoarthritis (249).) Also of mention is an experimental model in which an extensive subcutaneous striping lesion of the rat hind paw resulted in Wallerian degeneration of myelinated fibers of the involved spinal nerve (46), which again may be due to cPNL induced by pain-related postural changes. The latter factor may also have influenced the results of investigations that are cited in support of autonomous central sensitization (4).

Unfortunately, there is no animal model to demonstrate transition of acute to chronic pain. Furthermore, experiments may not deal with chronic pain as such, because the available pain assays (250, 251) do not necessarily indicate presence of persistent pain. Therefore, definitive testing of the introduced hypothesis should primarily involve multiple clinical studies targeted at diagnosing or excluding cPNL in chronic pain patients. The above explanations of the clinical chronic pain-related conundrums could serve as a rough logical testing of the hypothesis and help to develop relevant research. Obviously, clinical disorders, in which the cPNL hypothesis could be tested, are not limited to those discussed here.

## Conclusions

The current report offers a practical explanation of chronic pain. Differently from the previous concepts, this paper hypothesizes that there is a common neuropathic etiology of all types of general chronic pain, and that transition of acute to chronic pain may involve development (or aggravation) of peripheral compressive proximal neural lesion.

Described is a physioanatomical mechanism of persistent *ectopic peripheral neural* input that possibly maintains functional PNL in the form of DRGn hyperexcitability and its consequent hyperactivity (i.e., peripheral sensitization), which leads to hyperexcitability of central nociceptive pathway (i.e., central sensitization). Initial DRGn hyperexcitability, which can be induced by both peripheral non-neural and neural lesions, may produce an intermittent cPNL *via* reflexive myospasm-induced myofascial tension as well as *via* resultant muscle imbalance- and/or pain-provoked compensatory overuse. Consequently, DRGn hyperexcitability and cPNL may reciprocally maintain each other. Reciprocity may also exist between cPNL-caused motor fiber dysfunction and muscle weakness-related compensatory overuse. This vicious cycle seems to be catalyzed by sensitization (i.e., neuronal hyperexcitability) that increases nerve susceptibility to damage. Focal neuroinflammation induced by cPNL is a pathophysiological cause of DRGn hyperexcitability. Intra- and para-neural fibrosis can cause dynamic neural damage by restricting mobility of nerves. Intermittent nature of cPNL may be essential in chronic pain, because healed (fibrotic) lesions are physiologically silent to provide nociceptive input. Stimuli by distal lesions alone may be insufficient to maintain chronic pain and cannot be fueled by the vicious cycle. Pain in isolated conditions that are usually painless, as well as overly intense pain in inherently painful lesions, may be caused by mechanical hyperalgesia and allodynia due to compressive PNL-induced sensitization. Initially, cPNL may be asymptomatic until its aggravation or occurrence of additional focal or systemic nerve damage, i.e., development of a double crush-type injury. Not all patients may be equally susceptible to develop cPNL, because occurrence of cPNL may vary as vary patients' musculoskeletal fitness, exposure to overuse injury and past traumas, affliction by systemic diseases, and other factors. This article encourages diagnostic alertness to compressive PNL in chronic pain management and opens new perspectives for clinical and experimental research.

## Data Availability

The original contributions presented in the study are included in the article/**Supplementary Material**, further inquiries can be directed to the corresponding author.
