# Supplementary material for: Chronic pain and local pain in usually painless conditions including neuroma may be due to compressive proximal neural lesion
Source: Front Pain Res (Lausanne). 2023 Feb 20;4:1037376. doi: 10.3389/fpain.2023.1037376 (PMC9986610; doi:10.3389/fpain.2023.1037376)
Supplement: Supplementary file 1 [file Presentation1.pdf]

Supplementary Material (1)

(Article synopsis with overview figures and references)

Chronic pain and local pain in usually painless conditions including neuroma may be due to compressive proximal neural lesion

Valdas Macionis, MD, PhD
Correspondence: Valdas Macionis: valdas.macionis.md@gmail.com

Article synopsis

1. Prehypothetical, *ectopic* nociceptive sensitization-related reasoning

- ✓ *Acute pain may transform into chronic pain, i.e., pain that lasts after tissue insult has healed (or, conventionally, more than 3 months (1)).*
- ✓ *Acute pain is caused by painful (nociceptive) stimuli produced by noxious stimuli (i.e., noxious events such as tissue mechanical injury, heat, etc.). (The terms nociceptive and noxious stimuli are largely used interchangeably. See reference (2) for definitions.)*
- ✓ *Noxious stimuli provide nociceptive input that activates nociceptors, a subtype of peripheral afferents (primary sensory neural cells) in the dorsal root ganglion (DRG) (the term of nociceptor is also used as a synonym for pain receptor, i.e., the ending of the nociceptive fiber) (2–4).*
- ✓ *Nociceptors generate and transmit nociceptive neural impulses (action potentials) to the central nervous system (CNS) (Overview Fig. 1ov).*
- ✓ *Peripheral sensory cells can be activated ectopically, i.e., not only by noxious stimuli to receptors in the tissues, but also directly by stimulating the fibers or cell bodies of the afferent neurons (3,4) (Overview Fig. 2ovA).*
- ✓ *Certain stimulation of nociceptors may cause them to become sensitized (hyperexcitable and, consequently, hyperactive). This is peripheral sensitization: hyperexcitability and resultant hyperactivity (also understood as spontaneous ongoing discharge of neural impulses) of the peripheral sensory pathway (4–6).*
- ✓ *Experimental research indicates that one of the neurophysiological causes of chronic pain is ongoing (spontaneous) activity and mechanical sensitivity of sensory fibers (3,7,8), which results from their hyperexcitability (mechanosensitivity is a type of hyperexcitability).*
- ✓ *Hyperexcitability of sensory neurons enables continuing generation and discharge of neural impulses, which is necessary to be processed by brain cortex as persistent pain (i.e., chronic pain).*
- ✓ *Ongoing neuronal activity seems to be in part driven by neuroinflammatory mechanisms regardless of the triggering insult type (5,6).*
- ✓ *Neuroinflammation can be induced not only by systemic factors, but also by local neural injury, such as nerve compression (9).*
- ✓ *Nerve damage produces stronger sensitizing input than peripheral non-neural lesions: skin hypersensitivity lasts up to 24 hours after capsaicin injection (10), ten days after incision (11), and over two months after nerve injury (12).*
- ✓ *Peripheral sensory cells of any subtype can be sensitized ectopically (7), in particular by nerve compression, which induces local neuritis (9) and a resultant molecular/ionic cascade (3) that eventually causes neuron hyperexcitability and ongoing activity (Overview Fig. 2ovB).*
- ✓ *Peripheral sensitization causes central sensitization: hyperexcitability of the central sensory pathway (a converse process is also possible (5)), which can result in chronic pain (5,6) (Overview Fig. 2ovB).*
- ✓ *Sensitization enables noxious stimuli to cause hyperalgesia (abnormally extreme pain to noxious stimuli) (Overview Fig. 3ov) and non-noxious stimuli to cause painful sensations (allodynia) (Overview Fig. 4ov) (2), which is nociceptive hypersensitivity.*
- ✓ *Central sensitization enables hyperalgesia and allodynia in body regions that have not been affected by initial insult, which is widespread hypersensitivity (5).*

- ✓ *Pain generation and transmission involves interneuronal communication (crosstalk) both at the peripheral and central level, which is ananatomofunctional basis of allodynia and extraterritorial hypersensitivity (2,13) (Overview Fig. 4ov).*
- ✓ *Most chronic pain conditions are associated with central sensitization (14).*
- ✓ *Experimental research that supports autonomous central sensitization (i.e., occurring independently of peripheral sensitization) is relatively scanty (14) and is outweighed by contrary evidence (15,16).*
- ✓ *It has been shown that sensitization effects resolve without continuing focal neuroinflammatory input (7,8). This implies existence of occult peripheral neural damage in unexplained chronic pain.*
- ✓ *Occult development of peripheral neural lesion as a cause of chronic pain can also be supported by the studies that have revealed signs of peripheral neural damage after non-neural tissue injury (17-19).*
- ✓ *There is increasing evidence of neuropathic etiology of chronic pain in conditions commonly thought to be of non-neural origin. The unitary neural etiology of chronic pain is becoming particularly evident from the high prevalence of accompanying small fiber neuropathy (SFN) (20), the causes of which involve peripheral neural damage of various origin, including compressive neuropathies (21).*
- ✓ *Once a nerve trunk is compressed at one site, the nerve becomes more susceptible for compression at another level. In other words, nerve compression (and consequent sensitization) increases nerve vulnerability. In bifocal nerve compression, the resultant neurological deficit has been found to be greater than the sum of the deficits produced by each of the lesions individually (22). This is the double crush concept.*
- ✓ *Peripheral nerve injury results both in loss of DRG neurons (proximal injuries being more detrimental (23)) and in death of neurons in the dorsal horns of the spinal cord (24). Therefore, distal nerve damage may eventually generate a secondary (ectopic) proximal neural lesion (PNL) manifesting as structural and/or functional changes in the DRG and CNS.*
- ✓ *The closer the injury to the DRG, the more neurons are lost (23) and the greater overall excitation of DRG neurons can be effected via inflammatory processes, because more fibers are damaged in proximal nerve injuries than in distal ones (23). Similarly, Wallerian myelinated fiber degeneration, which induces spontaneous activity of the neighboring uninjured C-fibers (25) and thus contributes to sensitization, involves a longer length of the nerve distal to the damage (and consequently can produce greater cumulative sensitizing effect) in proximal than in distal nerve lesions.*

2. What has not been explained

2A. Why does acute pain transform into chronic pain?

- ✓ *In other words: Why do the nociceptive pathways appear to be ongoing hyperactive, even when there is no obvious tissue lesion that could provide nociceptive input?*
- ✓ *Another possible neurophysiological rephrasing of this question is as follows. If ectopic neuroinflammatory processes drive sensitization, then why do these processes persist, even when there is no obvious causative tissue lesion?*

2B. What is the mechanism of neural damage in non-neural tissue disorders with chronic pain?

2C. Why does chronic pain not afflict all patients with identical chronic pain-prone conditions?

3. What this article hypothesizes as an answer to the unexplained phenomena

- ✓ *If ongoing neuronal activity, maintained by neuroinflammation-related processes, drives chronic pain, then there should be occult ectopic sensitizing input in persistent pain without obvious tissue lesion.*
- ✓ *Transition of acute to chronic pain may involve development (or aggravation) of occult compressive peripheral proximal neural lesion (cPNL) in all types of general chronic pain.*

- ✓ *Most chronic pain conditions can be associated with musculoskeletal postural changes that may result in cPNL and consequent sensitization, which can drive a vicious cycle of chronic pain (Overview Fig. 5ov).*
- ✓ *Via pain and motor fiber damage, cPNL can worsen the causative musculoskeletal dysfunction, which may result in reciprocity between the latter two factors.*
- ✓ *Pathogenesis of the chronic pain cycle is as follows. Initial DRG neuron (DRGn) hyperexcitability (which can be induced by both peripheral non-neural and neural lesions) may produce an intermittent cPNL via reflexive myospasm-induced myofascial tension as well as via resultant muscle imbalance- and/or pain-provoked compensatory overuse. Consequently, DRGn hyperexcitability and cPNL may reciprocally maintain each other (Overview Fig. 5ov).*
- ✓ *Because of the latter reciprocity and relatively greater number of neurons involved, cPNL is more likely to maintain DRGn hyperexcitability in comparison to distal neural and non-neural lesions.*
- ✓ *Focal neuroinflammation induced by cPNL is a pathophysiological cause of DRGn hyperexcitability.*
- ✓ *According to the double crush concept, DRGn hyperexcitability probably catalyzes the chronic pain cycle: compressed (i.e., sensitized) nerves become susceptible to further damage.*
- ✓ *Pain in isolated conditions that are usually painless, as well as overly intense pain in inherently painful lesions, may be caused by hyperalgesia and mechanical allodynia due to cPNL-induced sensitization.*
- ✓ *Intra- and para-neural fibrosis can cause dynamic functional neural damage by restricting mobility of nerves, which is likely to occur in cPNL.*
- ✓ *Intermittent (dynamic) nature of cPNL may be essential in chronic pain, because healed (i.e., fibrotic) lesions are physiologically silent and, consequently, cannot provide nociceptive input.*
- ✓ *Neuroma can be an example of healed nerve injury that cannot act as permanent nociceptive input. Logically, as a local chronic pain condition, painful neuroma should have a source of nociceptive sensitization. Therefore, a reasonable explanation of neuroma pain could be coincidental cPNL-induced nociceptive sensitization (which involves ongoing activity and mechanosensitivity of afferent neurons). Notably, trauma is a common risk factor shared by neuroma and PNL.*
- ✓ *Not all patients may be equally susceptible to develop cPNL, because occurrence of cPNL may vary as vary patients' musculoskeletal fitness, exposure to overuse injury and past traumas, affliction by systemic diseases, and other factors.*
- ✓ *Development of pain provoked-postural changes and consequent cPNL in laboratory animals may influence experimental results.*
- ✓ *Having in mind the possibility of background compressive PNL due to negative musculoskeletal-postural impact can help to explain unclear aggravation and origin of chronic pain in many conditions, including spinal cord injury, fibromyalgia, and other systemic diseases.*
- ✓ *Dynamic nature and symptomatic complexity of proximal neural lesions may be the cause of frequent misdiagnosis of chronic pain.*

Overview References

1. Barke A, Korwisi B, Jakob R, Konstanjsek N, Rief W, Treede R-D. Classification of chronic pain for the International Classification of Diseases (ICD-11): results of the 2017 international World Health Organization field testing. *Pain* (2022) 163:e310–e318. doi: 10.1097/j.pain.0000000000002287
2. Sandkühler J. Models and mechanisms of hyperalgesia and allodynia. *Physiol Rev* (2009) 89:707–758. doi: 10.1152/physrev.00025.2008. PDF file available at https://journals.physiology.org/doi/epdf/10.1152/physrev.00025.2008
3. Gold MS. "Peripheral pain mechanisms and nociceptor sensitization.," In: Ballantyne JC, Fishman SM, Rathmell JP, editors. *Bonica's management of pain*. Lippincott Williams & Wilkins (LWW) (2018). p. 24–37. PDF file available at https://pcpr.pitt.edu/wp-content/uploads/2019/08/Bonica-Chapter-on-nociceptive-afferents.pdf
4. Woolf CJ, Ma Q. Nociceptors--noxious stimulus detectors. *Neuron* (2007) 55:353–364. doi: 10.1016/j.neuron.2007.07.016. PDF file available at https://www.cell.com/action/showPdf?pii=S0896-6273%2807%2900537-5

5. Ji R-R, Nackley A, Huh Y, Terrando N, Maixner W. Neuroinflammation and central sensitization in chronic and widespread pain. *Anesthesiology* (2018) 129:343–366. doi: 10.1097/ALN.0000000000002130
6. Matsuda M, Huh Y, Ji R-R. Roles of inflammation, neurogenic inflammation, and neuroinflammation in pain. *J Anesth* (2019) 33:131–139. doi: 10.1007/s00540-018-2579-4
7. Satkeviciute I, Goodwin G, Bove GM, Dilley A. Time course of ongoing activity during neuritis and following axonal transport disruption. *J Neurophysiol* (2018) 119:1993–2000. doi: 10.1152/jn.00882.2017. PDF file available at https://journals.physiology.org/doi/pdf/10.1152/jn.00882.2017
8. Greening J, Dilley A. "Peripheral mechanisms of chronic upper limb pain.," In: Fernandez de las Penas C, Cleland JA, Huijbregts PA, editors. *Neck and Arm Pain Syndromes*. Elsevier (2011). p. 476–495 doi: 10.1016/B978-0-7020-3528-9.00037-6
9. Rempel D, Dahlin L, Lundborg G. Pathophysiology of nerve compression syndromes: response of peripheral nerves to loading. *J Bone Joint Surg Am* (1999) 81:1600–1610. doi: 10.2106/00004623-199911000-00013. PDF file available at https://cutt.ly/02S8VrF
10. Arendt-Nielsen L, Andersen OK. "Capsaicin in human experimental pain models of skin, muscle and visceral sensitization.," In: Malmberg AB, Bley KR, editors. *Turning up the heat on pain: TRPV1 receptors in pain and inflammation*. Progress in Inflammation Research. Basel: Birkhäuser-Verlag (2005). p. 117–144 doi: 10.1007/3-7643-7379-2_7
11. Brennan TJ. Pathophysiology of postoperative pain. *Pain* (2011) 152:S33–S40. doi: 10.1016/j.pain.2010.11.005
12. Bennett GJ, Xie Y-K. A peripheral mononeuropathy in rat that produces disorders of pain sensation like those seen in man. *Pain* (1988) 33:87–107. doi: 10.1016/0304-3959(88)90209-6
13. Woolf CJ. Central sensitization: implications for the diagnosis and treatment of pain. *Pain* (2011) 152:S2–S15. doi: 10.1016/j.pain.2010.09.030. PDF file available at https://www.ncbi.nlm.nih.gov/pmc/articles/PMC3268359/pdf/nihms249521.pdf
14. Harte SE, Harris RE, Clauw DJ. The neurobiology of central sensitization. *J Appl Biobehav Res* (2018) 23:e12137. doi: 10.1111/jabr.12137
15. Baron R, Hans G, Dickenson AH. Peripheral input and its importance for central sensitization. *Ann Neurol* (2013) 74:630–636. doi: 10.1002/ana.24017
16. Brazenor GA, Malham GM, Teddy PJ. Can central sensitization after injury persist as an autonomous pain generator? A comprehensive search for evidence. *Pain Med Malden Mass* (2022) 23:1283–1298. doi: 10.1093/pm/pnab319
17. Orita S, Ishikawa T, Miyagi M, Ochiai N, Inoue G, Eguchi Y, Kamoda H, Arai G, Toyone T, Aoki Y, et al. Pain-related sensory innervation in monoiodoacetate-induced osteoarthritis in rat knees that gradually develops neuronal injury in addition to inflammatory pain. BMC Musculoskelet Disord (2011) 12:134. doi: 10.1186/1471-2474-12-134. PDF file available at https://bmcmusculoskeletdisord.biomedcentral.com/articles/10.1186/1471-2474-12-134
18. Ferreira-Gomes J, Adães S, Sousa RM, Mendonça M, Castro-Lopes JM. Dose-dependent expression of neuronal injury markers during experimental osteoarthritis induced by monoiodoacetate in the rat. Mol Pain (2012) 8:50. doi: 10.1186/1744-8069-8-50
19. Kajita Y, Suetomi K, Okada T, Ikeuchi M, Arai Y-CP, Sato K, Ushida T. Behavioral and neuropathological changes in animal models of chronic painful scar. J Orthop Sci (2013) 18:1005–1011. doi: 10.1007/s00776-013-0453-7. PDF file available at https://www.ncbi.nlm.nih.gov/pmc/articles/PMC3838578/
20. Chan ACY, Wilder-Smith EP. Small fiber neuropathy: Getting bigger! Muscle Nerve (2016) 53:671–682. doi: 10.1002/mus.25082
21. Schmid AB, Hailey L, Tampin B. Entrapment neuropathies: challenging common beliefs with novel evidence. J Orthop Sports Phys Ther (2018) 48:58–62. doi: 10.2519/jospt.2018.0603. PDF file available at https://www.jospt.org/doi/pdf/10.2519/jospt.2018.0603
22. Dellon AL, Mackinnon SE. Chronic nerve compression model for the double crush hypothesis. Ann Plast Surg (1991) 26:259–264. doi: 10.1097/00000637-199103000-00008
23. Hart AM, Terenghi G, Wiberg M. Neuronal death after peripheral nerve injury and experimental strategies for neuroprotection. Neurol Res (2008) 30:999–1011. doi: 10.1179/174313208X362479
24. Costigan M, Scholz J, Woolf CJ. Neuropathic pain: a maladaptive response of the nervous system to damage. Annu Rev Neurosci (2009) 32:1–32. doi: 10.1146/annurev.neuro.051508.135531
25. Wu G, Ringkamp M, Murinson BB, Pogatzki EM, Hartke TV, Weerahandi HM, Campbell JN, Griffin JW, Meyer RA. Degeneration of myelinated efferent fibers induces spontaneous activity in uninjured C-fiber afferents. J Neurosci (2002) 22:7746–7753.
26. Woolf CJ. Central sensitization: uncovering the relation between pain and plasticity. Anesthesiology (2007) 106:864–867. doi: 10.1097/01.anes.0000264769.87038.55. PDF file available at https://cutt.ly/p21FR7P
27. Campbell JN, Meyer RA. Mechanisms of neuropathic pain. Neuron (2006) 52:77–92. doi: 10.1016/j.neuron.2006.09.021.
PDF file available at https://www.cell.com/action/showPdf?pii=S0896-6273%2806%2900728-8

Overview Figures

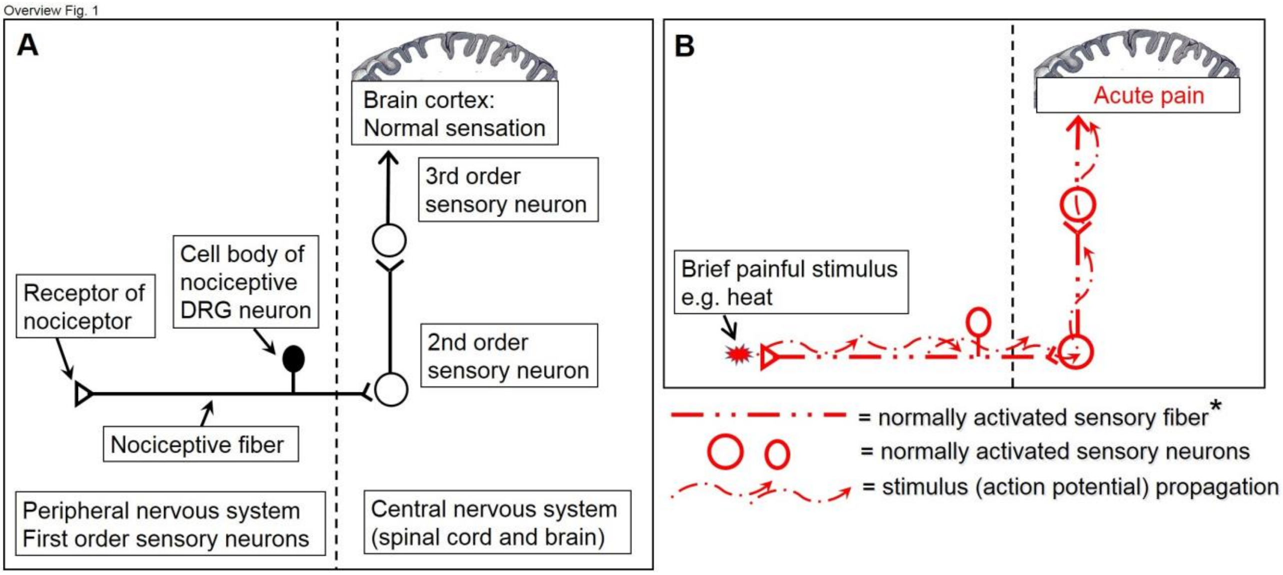

Overview Fig. 1ov. Simplified diagram of normal nociception. (**A**) Anatomic pathway of nociception. (**B**) Normal generation and perception of acute pain.
DRG = dorsal root ganglion
* diagrammatically represents a mixture of sensory neurons (both of A- and C-type), functional interplay of which is involved in producing complex neural symptomatics.

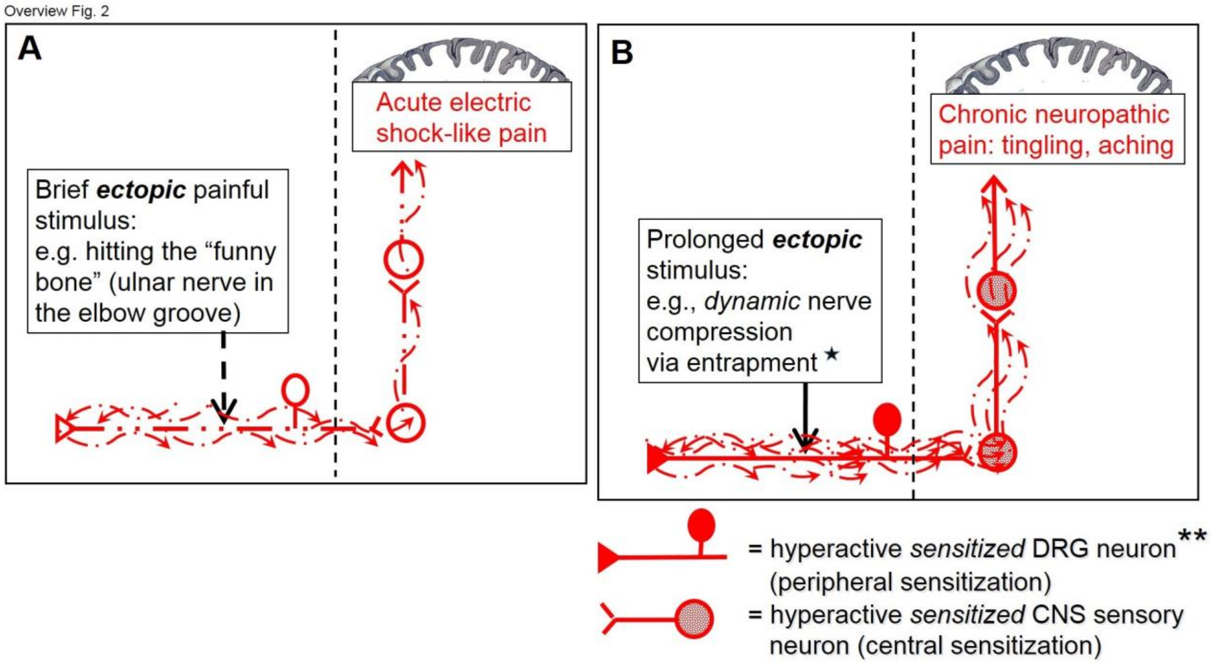

Overview Fig. 2ov. *Ectopic* generation of pain and sensitization. (**A**) Acute pain induction via ectopic stimulation of a peripheral nerve. Note *antidromic* excitation of the sensory fiber and its ending, which in the form of painful tingling is a common experience in hitting the "funny bone". (**B**) Neuropathic pain induction via ectopic neural lesion that causes sensitization.
For pictogram descriptions, see also the legend of Overview Fig. 1ov.
*The resultant focal neuroinflammation, which is a pathophysiological cause of sensitization, is not illustrated here (this is shown in Supplement Figs. 1S. 2S, and 4S and in the main article Fig. 1).
** diagrammatically represents a mixture of sensory neurons (both of A- and C-type), functional interplay of which is involved in producing complex neural symptomatics.

Overview Fig. 3

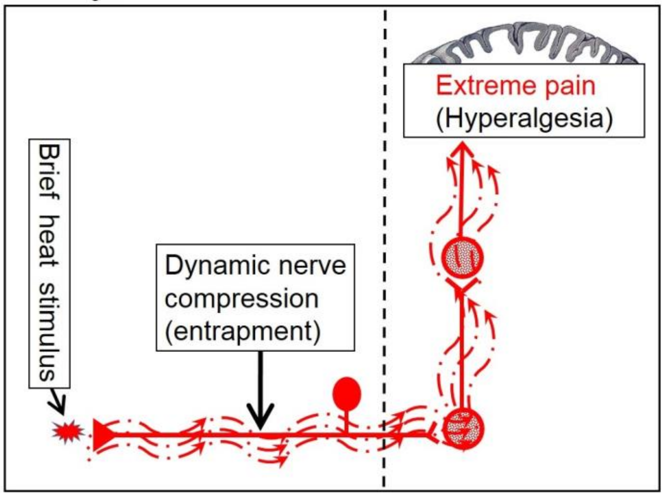

Overview Fig. 3ov. Simplified diagram of the mechanism of hyperalgesia, induced via *ectopic* sensitization. See [Overview Fig. 4ov] below and Fig.1 in the main article for further examples of ectopically induced local hypersensitivity.
For pictogram descriptions, refer also to the legend of Overview Fig. 1ov and Overview Fig. 2ovB.

Overview Fig. 4

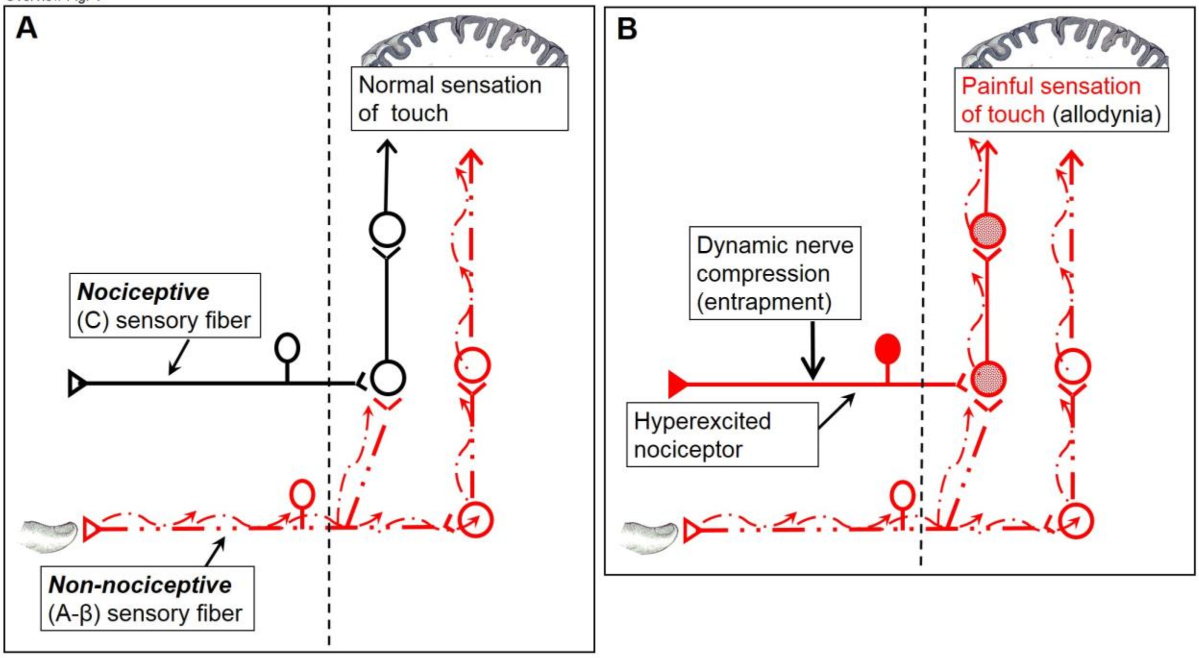

Overview Fig. 4ov. Simplified diagram of induction of allodynia via *ectopic* sensitization. (**A**) Induction of normal sensation of touch. Note that the *intraspinal collateral* postganglionic fiber of the non-nociceptive neuron is not able to convey impulses to the nociceptive pathway, which is thought to be due to synaptic inhibition and other mechanisms (2). (**B**) Touch results in painful sensation because of sensitization of the nociceptive pathway and intraspinal neuronal crosstalk that enables the relay of the non-nociceptive touch-generated impulses to the nociceptive route. This is now possible because of synaptic disinhibition (26) and other mechanisms due to central sensitization (not shown). A simple diagrammatic explanation of central spinal sensitization can be found in Woolf (13,26) and Campbell and Meyer (27)). See Overview Fig. 2ovB for graphic explanation of ectopic sensitization.
For pictogram descriptions, refer also to the legend of Overview Fig. 1ov and Overview Fig. 2ovB.

Overview Fig. 5

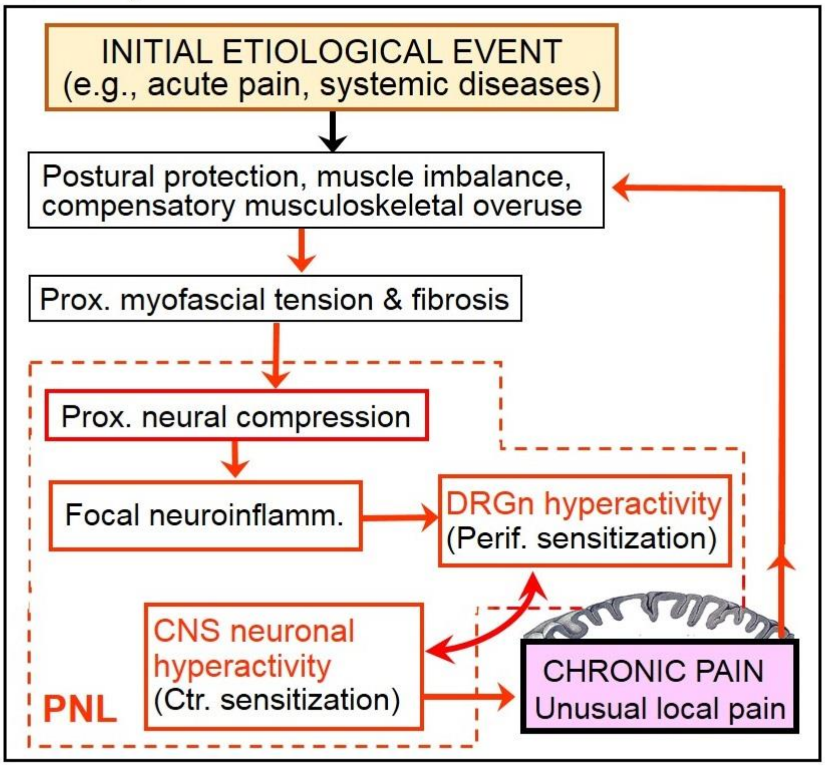

Overview Fig. 5ov. Simplified hypothetical mechanism of the vicious cycle of chronic pain. A more comprehensive version is presented in the main article Fig. 2**.** Note that neuronal hyperactivity is a result of neuronal hyperexcitability (i.e., sensitization).

Redlined boxes = major drivers of the vicious cycle; violet filled-in box = processing of nociceptive impulses by the brain cortex; black flow arrow = triggering input which is not permanently involved in the vicious cycle; red flow one-headed arrow = input which serves as a link of the vicious cycle; bowed double-headed arrow = reciprocal enhancement but *not an independent cycle*.

Prox. = Proximal; neuroinflamm. = neuroinflammation; DRGn = dorsal root ganglion neuron; Perif. = Peripheral; PNL = proximal neural lesion; CNS = central nervous system; Ctr. = Central.
