# Supplementary material for: Chronic pain and local pain in usually painless conditions including neuroma may be due to compressive proximal neural lesion
Source: Front Pain Res (Lausanne). 2023 Feb 20;4:1037376. doi: 10.3389/fpain.2023.1037376 (PMC9986610; doi:10.3389/fpain.2023.1037376)
Supplement: Supplementary file 3 [file Presentation3.pdf]

Supplementary Material (3)

(Supplement figures 1S, 2S, 3S, and 4S)

Chronic pain and local pain in usually painless conditions including neuroma may be due to compressive proximal neural lesion

Valdas Macionis, MD, PhD
Correspondnce: Valdas Macionis: valdas.macionis.md@gmail.com

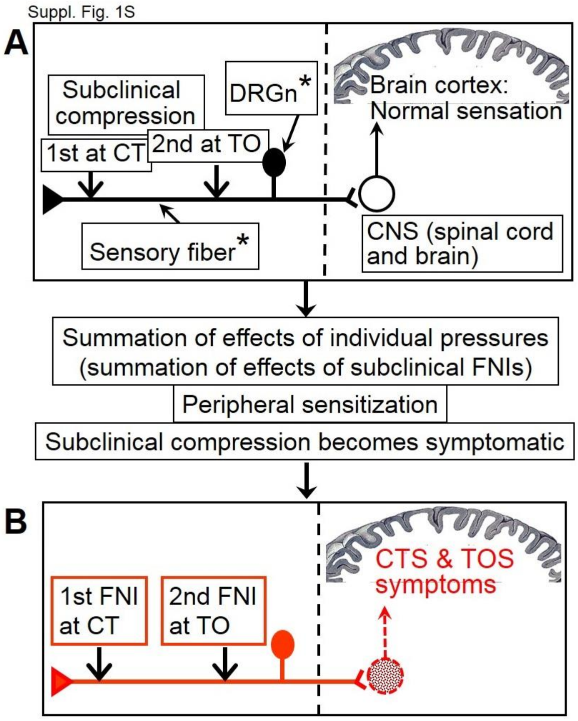

Supplement Fig. 1S. Simultaneous nerve compression by two forces, the individual effects of which are asymptomatic (**A**), may induce nociceptive peripheral sensitization and consequent double crush syndrome, which here clinically presents as CTS and TOS (B). Note the hyperexcited peripheral nociceptive pathway, which is due to cumulative effect of focal neuroinflammations induced by double compression (**B**).

Filled in triangle = receptor of the sensory neuron; black vertical down arrow over the sensory fiber = force that does not produce symptomatic compression of otherwise unaffected nerve; black triangle with black horizontal line and black DRGn = peripheral sensory pathway in a normal state; red triangle with red horizontal line and red DRGn = hyperexcited peripheral sensory pathway; white circle = neuron of the dorsal horn of the spinal cord (arrow shows impulse direction); red pattern-filled circle = hyperactive dorsal horn neuron (arrow shows impulse direction).

DRGn = dorsal root ganglion neuron body; CT = carpal tunnel; TO = thoracic outlet; CNS = central nervous system; FNI = focal neuroinflammation; CTS = carpal tunnel syndrome; TOS = thoracic outlet syndrome.

* diagrammatically represents mixture of sensory neurons (both of A- and C-type), which are involved in producing complex symptomatics of neural lesion. The complex involvement of central sensitization and neuron crosstalk in the DRG and CNS is not shown (simple diagrammatic explanations of central sensitization are presented in the supplement file “Overview” Overview Fig. 4ov, in Woolf (1), and Campbell and Meyer (2)).

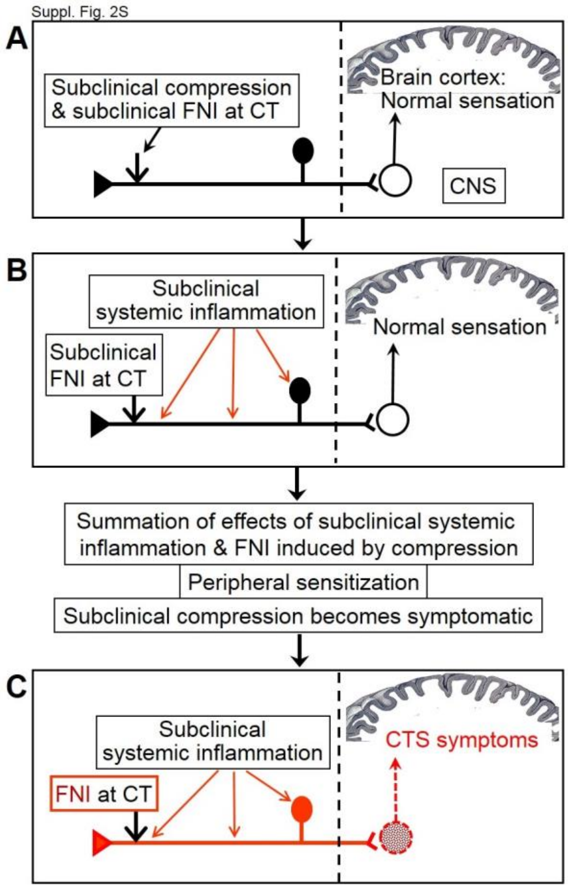

Supplement Fig. 2S. Hypothetical mechanism of peripheral sensitization induced by summation of effects of subclinical nerve compression, i.e., subclinical focal neuroinflammation (**A**) and systemic neuroinflammation (**B** and **C**). The subclinical compression resulted in CTS because of consequent nerve trunk sensitization (**C**).
For abbreviations and pictogram descriptions, refer to the legend of Supplement Fig. 1S.

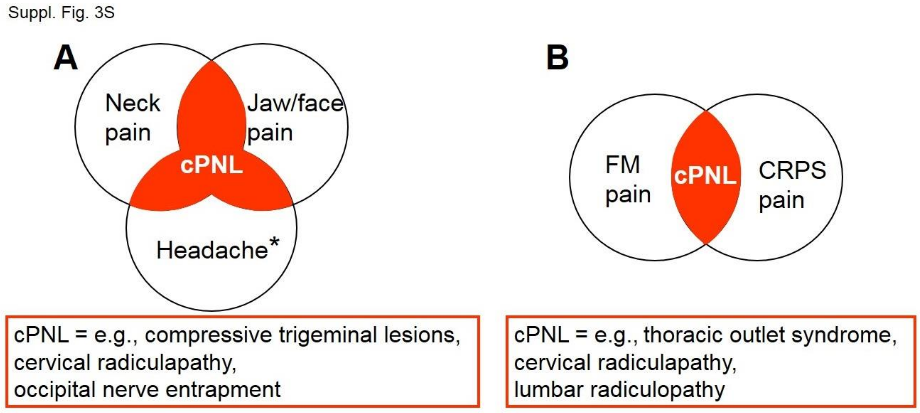

Supplement Fig. 3S. Compressive PNL as a hypothetical etiology of overlapping chronic pain. Combinations of multiple-site cPNL are possible. Adapted from Maixner et al. (3)
cPNL = compressive proximal neural lesion; FM = fibromyalgia; CRPS = complex regional pain syndrome.
*excludes organic causes, e.g., intracranial hypertension and the true migraine.

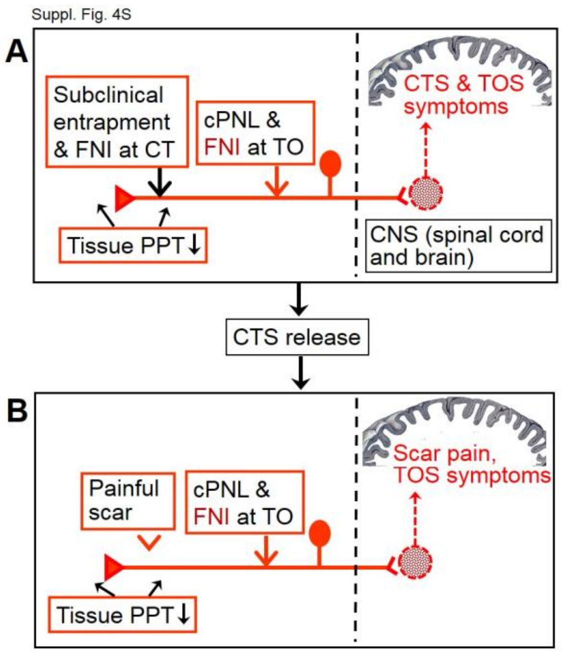

Supplement Fig. 4S. Hypothetical mechanism of painful scar development due to residual peripheral sensitization maintained by unreleased proximal nerve compression in double crush syndrome. Subclinical distal compression manifests as CTS because of nerve trunk sensitization induced by cPNL, which here manifests as TOS (**A**). Unresolved peripheral tissue sensitization, accompanied by PPT decrease, causes scar pain (**B**). Note that DRGn hyperexcitability is primarily due to TOS-induced focal neuroinflammation. Differently from the double crush syndrome version shown in Supplement Fig. 1S, CTS here results from the sensitizing effects of TOS only, rather than from cumulative effect of both disorders.
Red tick = painful scar at the site of carpal tunnel release.
cPNL= compressive proximal neural lesion; PPT↓ = decrease of pressure pain threshold.
For other abbreviations and pictogram descriptions, refer to the legend of Supplement Fig. 1S.

References

1. Woolf CJ. Central sensitization: implications for the diagnosis and treatment of pain. Pain (2011) 152:S2–S15. http://www.rosslab.neurobio.pitt.edu/wp-content/themes/rosslab-theme/files/article_woolf.pdf
2. Campbell JN, Meyer RA. Mechanisms of neuropathic pain. Neuron (2006) 52:77–92. https://www.cell.com/neuron/pdf/S0896-6273(06)00728-8.pdf
3. Maixner W, Fillingim RB, Williams DA, Smith SB, Slade GD. Overlapping chronic pain conditions: implications for diagnosis and classification. *J Pain*. (2016) 17(9 Suppl):T93-T107.
